# Tartronic Acid as a Potential Inhibitor of Pathological Calcium Oxalate Crystallization

**DOI:** 10.1002/advs.202400642

**Published:** 2024-04-22

**Authors:** Yuan Su, Si Li, Xin Li, Jing‐Ying Zhou, Vraj P. Chauhan, Meng Li, Ya‐Hui Su, Chun‐Mei Liu, Yi‐Fei Ren, Wu Yin, Jeffrey D. Rimer, Ting Cai

**Affiliations:** ^1^ Department of Pharmaceutics China Pharmaceutical University Nanjing 211198 China; ^2^ Department of Pharmaceutical Engineering China Pharmaceutical University Nanjing 211198 China; ^3^ Department of Chemical and Biomolecular Engineering University of Houston Houston TX 77204 USA; ^4^ The State Key Lab of Pharmaceutical Biotechnology, College of Life Science Nanjing University Nanjing 210036 China

**Keywords:** calcium oxalate, crystal growth inhibitors, kidney stones, pathological mineralization

## Abstract

Kidney stones are a pervasive disease with notoriously high recurrence rates that require more effective treatment strategies. Herein, tartronic acid is introduced as an efficient inhibitor of calcium oxalate monohydrate (COM) crystallization, which is the most prevalent constituent of human kidney stones. A combination of in situ experimental techniques and simulations are employed to compare the inhibitory effects of tartronic acid with those of its molecular analogs. Tartronic acid exhibits an affinity for binding to rapidly growing apical surfaces of COM crystals, thus setting it apart from other inhibitors such as citric acid, the current preventative treatment for kidney stones. Bulk crystallization and in situ atomic force microscopy (AFM) measurements confirm the mechanism by which tartronic acid interacts with COM crystal surfaces and inhibits growth. These findings are consistent with in vivo studies that reveal the efficacy of tartronic acid is similar to that of citric acid in mouse models of hyperoxaluria regarding their inhibitory effect on stone formation and alleviating stone‐related physical harm. In summary, these findings highlight the potential of tartronic acid as a promising alternative to citric acid for the management of calcium oxalate nephropathies, offering a new option for clinical intervention in cases of kidney stones.

## Graphical abstract

This study introduces tartronic acid as a potent inhibitor of calcium oxalate monohydrate (COM) crystallization, pivotal in kidney stone formation, through specific inhibitor‐crystal interactions. In vivo experiments demonstrate the efficacy of tartronic acid in inhibiting stone formation and mitigating stone‐related harm in hyperoxaluria mouse models. This discovery holds significant promise for advancing the treatment of pathological calcium oxalate crystallization.

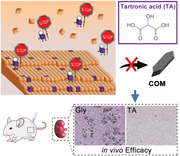

## 1 Introduction

Undesired crystallization within the human body can lead to various diseases, including kidney stones, gout, atherosclerosis, and arthritis.^[^ ^1^ ^]^ The kidneys are particularly susceptible to stone formation due to factors that lead to high mineral supersaturation and consequent crystallization, ultimately causing the formation of kidney stones.^[^ ^1^, ^2^ ^]^ There are many inorganic and organic compunds that lead to pathological crystallization in urine, with calcium oxalate monohydrate (COM) being the most prevalent constituent of kidney stones, accounting for 40–80% of cases.^[^ ^1^, ^3^ ^]^ Individuals suffering from kidney stones are at risk of a wide range of abnormalities, including acute kidney injury, chronic kidney disease, and renal fibrosis.^[^ ^4^ ^]^ Notably, the global prevalence of kidney stones is on the rise, exacerbated by lifestyle changes and global warming, with a recurrence rate of up to 50% within the first 5 years after the initial stone episode; however, non‐surgical treatment and prevention options remain exceedingly limited.^[^ ^1^, ^3^, ^5^ ^]^

The disruption of crystal growth by inhibitor‐crystal interactions has garnered significant attention in the pursuit of new therapies for preventing pathological crystallization.^[^ ^6^ ^]^ Inhibitors comprise a broad class of ions, molecules, and macromolecules that bind to specific crystal surface sites, including kinks, steps, and terraces.^[^ ^6^, ^7^ ^]^ These interactions impede solute incorporation through distinct mechanisms, which effectively reduce the rate of crystal growth. Previous research has uncovered a diverse number of COM crystal growth inhibitors, ranging from small molecules such as citrate derivatives, myo‐inositol hexakisphosphate analogs, and natural polyphenols to macromolecules like anionic proteins and glycosaminoglycans.^[^ ^6^, ^7^, ^8^ ^]^ Although myo‐inositol hexakisphosphate and natural polyphenols have demonstrated efficacy in a mouse model of hyperoxaluria, and the circulating protein AIM (apoptosis inhibitor of macrophage) has been found to dramatically suppress kidney stone development by impeding the aggregation and growth of COM crystals, the majority of these studies have focused on investigating how inhibitors affect crystal morphology or growth in vitro, with limited evidence of their activity in vivo.^[^ ^8^ ^]^ Consequently, the feasibility of utilizing crystal growth inhibitors as a therapeutic approach for calcium oxalate (CaOx) kidney stone diseases has remained underexplored.

Potassium citrate, the primary treatment for CaOx kidney stones, has been proven effective in preventing kidney stone formation by acting as a COM crystal inhibitor.^[^ ^6^, ^7^ ^]^ However, its associated gastrointestinal side effects often result in a low adherence rate.^[^ ^5^, ^9^ ^]^ Prior studies have revealed that molecules decorated with carboxylic acids are effective inhibitors of COM crystallization due to their ability to bind to the COM crystal surface through a calcium bridge, i.e., _(inhibitor)_COO^−^∙∙∙Ca^2+^∙∙∙^−^OOC_(Ox, COM)_.^[^ ^6^, ^7^, ^10^ ^]^ While the efficacy of these inhibitors generally correlates with the number of negative charges present in the molecule, recent research has unveiled the crucial role of the number and placement of hydroxyl groups in the interaction with COM surfaces.^[^ ^6^, ^7^, ^8^, ^10^, ^11^ ^]^ Tatronates, a series of compounds with hydroxy bis acid units, have demonstrated a binding affinity for hydrooxyapatite, a calcium‐based crystal found in bone.^[^ ^12^ ^]^ Among them, tartronic acid, the simplest hydrodicarboxylic acid, exhibits numerous pharmacological actions, including the inhibition of carbohydrates conversion and lactate dehydrogenase activity.^[^ ^13^ ^]^ In addition, tartronic acid demonstrated significant renal excretion when administered orally or by injection, without undergoing metabolism.^[^ ^14^ ^]^ These discoveries led us to hypothesize that this compound could serve as a promising renal CaOx inhibitor.

**Figure 1:**
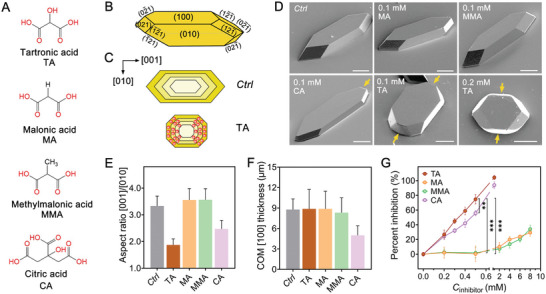
Effect of inhibitors on COM crystallization. A) Molecular structures of TA, MA, MMA, and CA. B) Illustration of COM crystal habit with indexed faces. C) Illustrations of the COM crystal habit and its modification by TA‐induced growth inhibition of apical faces. D) SEM images of COM crystals in the absence of inhibitor (control, *Ctrl*) and in the presence of 0.1 mm MA, 0.1 mm MMA, 0.1 mm CA, 0.1 mm TA, and 0.2 mm TA. All scale bars equal 20 µm. The (001) surface that develops in the presence of CA and TA is labeled with yellow arrows. E) Changes in COM [001]/[010] aspect ratio at inhibitor concentration, *C*_inhibitor_ = 0.1 mm. *n *= 158 *Ctrl*, *n *= 177 TA, *n *= 180 MA, *n *= 173 MMA, *n *= 169 CA. Each bar represents the average from three separate batches. F) Comparison of COM [100] average thickness at *C*_inhibitor_ = 0.1 mm. *n *= 26 *Ctrl*, *n *= 38 TA, *n *= 36 MA, *n *= 27 MMA and CA. Each bar represents the average from at least two separate batches. G) Percent inhibition of COM crystal growth as a function of *C*_inhibitor_. A mimimum of four measurements were conducted for each data point. *p < 0.05, **p < 0.01, ***p < 0.001 by two‐tailed one‐way ANOVA. Error bars equal one standard deviation in panels E and F and span two standard deviations in panel G. Source data are provided in the Source Data File.

In this work, we introduce tartronic acid (TA) as an efficient inhibitor of CaOx crystallization. The inhibitory effects of TA, along with its molecular analogs, malonic acid (MA) and methylmalonic acid (MMA), were assessed against those of citric acid (CA) (possessing three ‐COOH groups). Despite their structural similarities (**Figure** **1**A), these compounds display distinct inhibition potencies and binding preferences. Here we employ a combination of in situ analytical techniques, including microfluidic assays and atomic force microscopy (AFM), to monitor COM crystallization in real‐time at macroscopic and microscopic length scales, respectively. Our findings reveal that inhibitor‐crystal interactions constitute the predominant mechanism behind COM growth inhibition. Furthermore, the inhibitory effects of TA and its analogs in a human urine assay are assessed, indicating that TA is more effective in inhibiting COM crystallization compared to CA. Moreover, the effectiveness of TA was confirmed in a mouse disease model, suggesting its potential therapeutic activity on calcium oxalate nephrolithiasis. Collectively, our findings offer significant implications for the development of kidney stone therapies based on crystallization inhibition.

## 2 Results

### 2.1 Effect of Inhibitors on the Crystallization of COM Crystals

We examined the crystallization of COM in the presence of one tricarboxylic acid, citric acid (CA), and three dicarboxylic acids with similar structures (Figure 1A): tartronic acid (TA), malonic acid (MA), and methylmalonic acid (MMA). Figure 1B provides an illustration of the crystal habit of COM, which is bounded by the (100) basal surface, (010) side surfaces, and {021} and {12$\bar{1}$} faces comprising the apical tips.^[^ ^6^ ^]^ Our observations reveal that TA specifically alters the growth of COM crystals in the [001] direction (as illustrated in Figure 1C). In the presence of TA, the apical tips of the COM crystals become blunted (Figure 1D; Figures S1A and S4, Supporting Information), leading to decreased crystal length and a significant 43.5% reduction in the [001]/[010] aspect ratio at 0.1 mm TA (Figure 1E). Notably, at a TA concentration of 0.2 mm, the (010) surface disappeared (Figure 1D; Figures S1A and S5, Supporting Information). However, the presence of TA did not alter crystal thickness (Figure 1F), suggesting a specificity of TA to bind to the apical tips, which creates (001) faces that are typically absent in aqueous conditions due to kinetic factors.^[^ ^15^ ^]^ As the TA concentration is increased to 1.0 mm, COM crystallization is completely arrested (Figure S1A, Supporting Information). In contrast, the constant morphology of COM crystals formed in the presence of different concentrations of MA and MMA (Figure 1D; Figures S1 and S2, Supporting Information) indicates the minimal impact of MA and MMA on the crystallization of COM. To understand the effects of TA on COM crystallization, we repeated earlier studies using CA, which is known to inhibit growth in both the [001] and [100] directions, leading to the formation of thin crystals with blunted apical tips (Figure 1D; Figures S1 and S2, Supporting Information).^[^ ^6^, ^10^ ^]^ In the presence of 1.0 mm CA, despite a low crystal number density, COM continues to crystallize, indicating that CA is a less effective inhibitor than TA.

Kinetic studies using an ion selective electrode (ISE) to monitor the temporal depletion of free Ca^2+^ ions in a supersaturated calcium oxalate solution confirmed that TA is a more potent inhibitor of COM crystallization. At concentrations of 0.5 mm for both TA and CA, we observed 74.8 and 55.8% inhibition of COM crystallization, respectively (Figure 1G; Figure S6A,B, Supporting Information). As the concentration is increased to 1.0 mm, both inhibitors effectively suppressed COM crystal growth, with only a marginal depletion of free calcium observed within the experimental timeframe (Figure S6A,B, Supporting Information). In contrast, MA and MMA have a relatively weak effect on the kinetics of bulk COM crystallization (Figure 1G; Figure S6C,D, Supporting Information), with neither molecule exceeding 35% inhibition even at high concentrations (e.g., 8 mm). Crystal growth inhibitors typically operate through two mechanisms: solute complexation to reduce supersaturation and surface binding to impede solute incorporation. To ascertain whether inhibitor‐solute complexation is the dominant mode of COM growth inhibition, we monitored changes in the concentration of free Ca^2+^ ions in the presence of each inhibitor at different concentrations (Figure S7, Supporting Information). These measurements showed that the complexation between TA and Ca^2+^ at experimental concentrations is minimal, with only 13% of Ca^2+^ bound at 1.0 mm TA, contrasting significantly with CA, which sequesters about half of the free Ca^2+^ at the same concentration. This suggests that TA primarily inhibits COM crystallization through interactions with crystal surfaces, rather than forming complexes with Ca^2+^ in solution.

### 2.2 Microfluidic Analysis of COM Growth Inhibition

**Figure 2:**
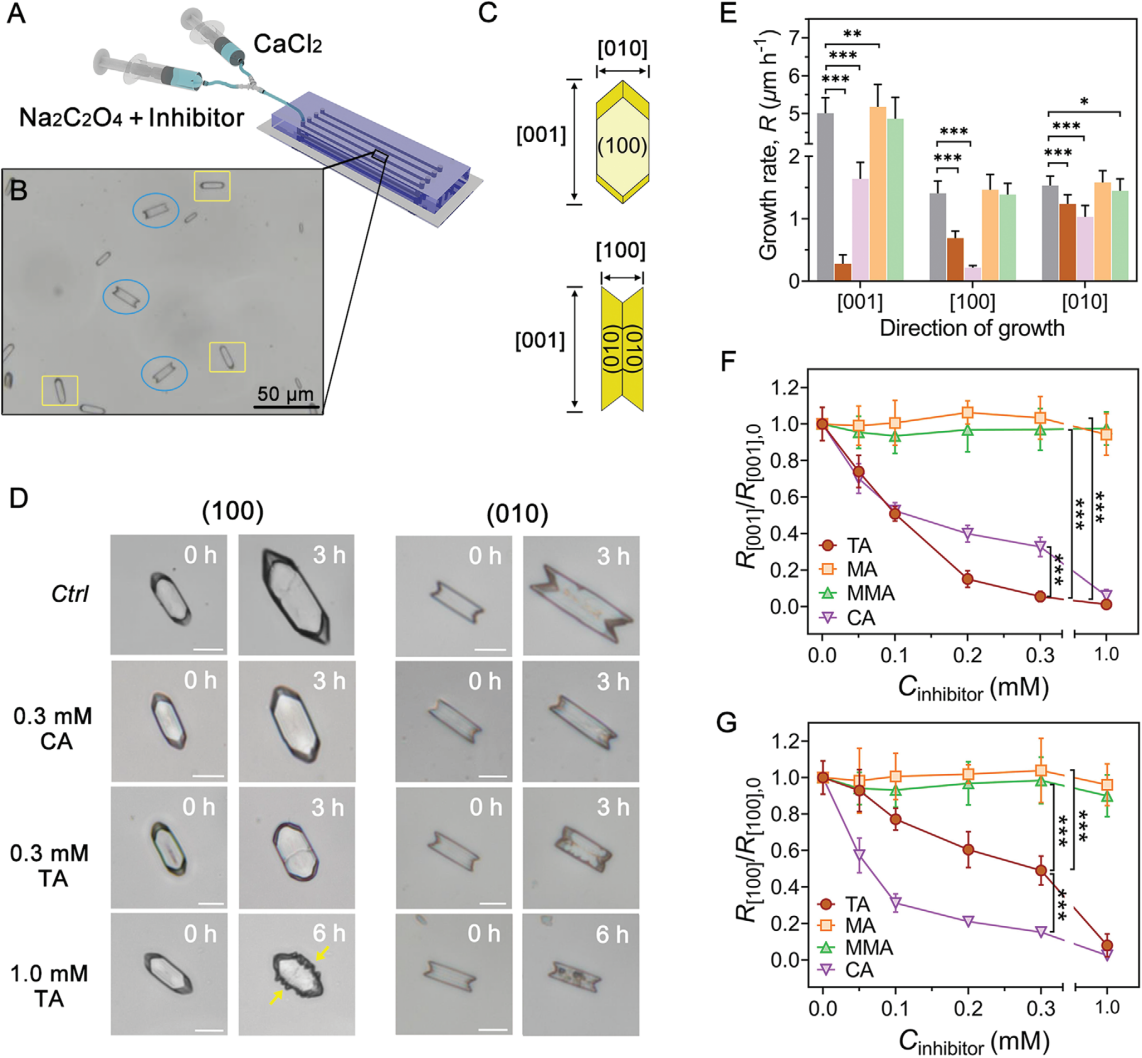
Effect of inhibitors on COM crystal growth under flow conditions. A) Schematic of the microfluidic device used for COM crystallization. B) Representative optical micrograph of COM seeds oriented along the microchannels in one of two configurations, with their faces normal to either the [100] (in yellow squares) or [010] (in blue cycles) directions. C) Schematic representation of the COM crystal with indexed facets. D) Time‐resolved optical micrographs of COM growth under constant flow (0.5 ml h^−1^) of supersaturated solutions (*S* = 3.6) in the absence and presence of 0.3 mm CA, 0.3 mm TA, and 1.0 mm TA (from top to bottom). All scale bars equal 10 µm. The roughness of (010) surfaces in the presence of 1.0 mm TA is highlighted with yellow arrows. E) Growth rate of COM crystals for all three principal crystallographic directions, [001] (*n* ≥ 58, at least five independent experiments), [100] (*n* ≥ 16, one or two independent experiments), and [010] (*n* ≥ 41, at least three independent experiments), in the absence and presence of 0.3 mm TA (gray), CA (violet), MA (brown), and MMA (orange). F,G) Relative growth rate in the [001] (F) and [100] (G) directions of COM crystals as a function of inhibitor concentration. Symbols in panel F are the averages of 58–166 crystals from at least six independent experiments, and symbols in panel G are the averages of at least 15 twin crystals from one to three independent experiments. Error bars equal one standard deviation in panel E and span two standard deviations in panels F and G. Data were all analyzed by two‐tailed one‐way ANOVA. *p < 0.05, **p < 0.01, and ***p < 0.001. Source data are provided in the Source Data File.

To quantitatively assess the selectivity of inhibitor interactions with COM crystals, a microfluidic device coupled with optical microscopy (**Figure** **2**A) was designed for real‐time observation of COM growth. COM seeds, oriented along the microchannels in one of two configurations with their faces normal to either the [100] or [010] directions, were generated within polydimethylsiloxane (PDMS) chips in situ (Figure 2B; Figure S8, Supporting Information) using two methods (see Experimental Section for details). This enabled real‐time monitoring of COM growth along all three principal crystallographic directions (Figure 2C). The crystals oriented along the [010] direction are characteristic of (100) contact penetration twins.^[^ ^15^ ^]^ The growth solutions used for microfluidic measurements were prepared with a supersaturation ratio of *S* = 3.6 using a molar composition of 0.5 mm CaCl_2_, 0.5 mm Na_2_C_2_O_4_, 150 mm NaCl, and *C*_inhibitor_ mm inhibitor (*C*_inhibitor_ = 0–2.0 mm). We observed that the growth rate of COM crystals along specific directions remained nearly constant regardless of the nucleation method and whether the crystals were single or twin crystals (Figure S9, Supporting Information); therefore, the growth rate along the [001] direction in Figure 2F is the average calculated from over 60 crystals, encompassing both single and twin crystals.

When TA was introduced in the growth solution, the apical tips of COM crystals in the microchannels became blunted over time (Figure 2D; Figure S10A, Supporting Information), consistent with the morphology change observed in quiescent bulk assays. As the TA concentration increased, the growth rate along the crystal length ([001] direction) decreased rapidly, with 0.3 mm TA reducing the rate by ≈93% (Figure 2F). TA also exhibited an inhibitory effect on growth along the [100] direction, impacting crystal thickness (Figure 2G; Figure S11A, Supporting Information). Notably, the presence of 0.3 mm TA resulted in a 50% reduction in [100] growth; however, this effect was relatively weak at low TA concentrations, resulting in no significant change in COM thickness in the quiescent bulk assays. In contrast, growth along the [010] direction (crystal width) was maintained at TA concentrations up to 0.3 mm (Figure S12, Supporting Information). The reduction in the growth profile followed the order [001] > [100] > [010] (Figure 2E), thus confirming the binding specificity of TA for the apical surfaces of COM crystals. When the TA concentration was increased to 1.0 mm, a significant roughness with the emergence of protrusions on (010) surfaces was observed (Figure 2D; Figures S13 and S14, Supporting Information). This is accompanied by a considerable decline in growth along the [010] direction, exceeding 40% inhibition (Figure S12, Supporting Information), while growth along the [001] and [100] directions is nearly arrested at such high inhibitor concentration. The appearance of protrusions on the (010) surfaces was unusual and similar to a previously reported feature of crystal whiskers that form at low concentrations of polyphenols due to the surface growth mechanism shifting from spiral dislocation to 2D nucleation, which are putatively associated with the alcohol moieties of polyphenols.^[^ ^7e^ ^]^ In our study, these protrusions only formed at high concentrations of TA, and the growth rate of protrusions decreased with increasing TA concentration (Figure S13, Supporting Information). Conversely, the molecular analogs MA and MMA exhibited almost no inhibition of COM growth in all three crystallographic directions (Figure 2F,G; Figure S12, Supporting Information). Additionally, the effect of 0.3 mm CA in the microfluidic assay revealed an 80% reduction in growth along the thickness ([100] direction), a 65% reduction in growth along the crystal length ([001] direction), and a 30% inhibition along the width ([010] direction). This suggests that CA primarily interacts with the (100) surfaces, consistent with previous results.^[^ ^6^, ^7^ ^]^ When 1.0 mm CA was introduced, crystal growth was completely suppressed along the [100] and [001] directions, and there was an almost 80% reduction along the [010] direction while the (010) surfaces remained smooth (i.e., absent of features observed in the presence of TA). The predicted relative growth rates based on the complexation effect (Figure S15, Supporting Information) further indicate that the inhibitory efficacy of TA on COM crystal growth primarily results from a kinetic effect, rather than a reduced supersaturation due to the complexation of free Ca^2+^ in the solution.

### 2.3 Molecular Mechanism of COM Growth Inhibition

**Figure 3:**
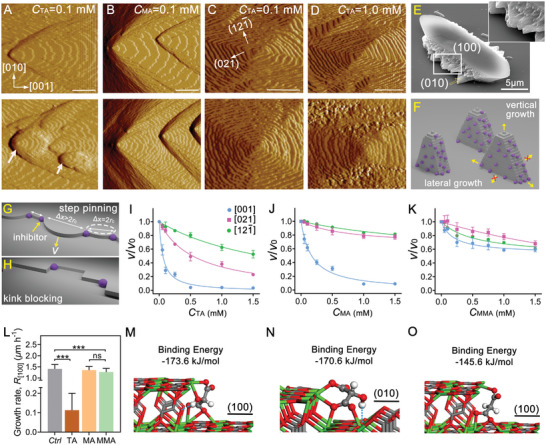
Impact of TA analogues on COM surface growth. A–D) AFM deflection mode images of COM (A,B) (100) and (C,D) (010) surfaces during in situ measurements in a supersaturated solution (*S* = 4.1) with 0.1 mm TA, 0.1 mm MA, and 1.0 mm TA. Top images were captured at initial scanning times and those on the bottom were taken after ≈13 min of continuous imaging. Arrows in panel A highlight rounded hillocks on the COM (100) surface in the presence of TA. Scale bars equal 1 µm in panels A and B and 0.5 µm in panels C and D. E) SEM image of a COM crystal grown in a microfluidic channel after 6 h in the presence of 1.0 mm TA. Protrusions formed on the (010) surface. F) Idealized representation of protrusions formed on (010) surfaces at high TA concentration. G,H) Illustrations of (G) step pinning and (H) kink blocking mechanisms of growth inhibition. I–K) Step velocity *v* in the presence of inhibitor scaled by the control in the absence of inhibitor, *v*_0_, as a function of (I) TA, (J) MA, and (K) MMA concentration. Green, magenta, and blue circles represent step advancement in the [12$\bar{1}$], [021], and [001] directions, respectively. Symbols are the average of at least five measurements, and error bars span two standard deviations. Lines are interpolated to guide the eye. L) Growth rate *R*_[001]_ of COM crystals in microfluidic channels along the [100] direction in the absence and presence of 1.0 mm TA, MA, and MMA. The experimental method employed is consistent with that described in Microfluidic analysis of COM growth inhibition. Error bars equal one standard deviation. *n* = 60 *Ctrl*, *n* = 38 TA, *n* = 32 MA, and *n* = 31 MMA from at least two independent experiments. Student's t‐test and one‐way ANOVA were used. *p < 0.05, **p < 0.01, ***p < 0.001. M,O) Side views and binding energies of stable structural conformations from DFT calculations of deprotonated (M) TA and (O) MA molecule adsorption at the [001] acute step on the (100) plane. N) Similar view of a TA molecule adsorbed at (021) step on the (010) plane. Atoms are colored to represent hydrogen (white), carbon (gray), oxygen (red), and calcium (green). Source data are provided in the Source Data File.

To provide a molecular‐level understanding of interactions between inhibitors and COM crystals, a combination of atomic force microscopy (AFM) and density functional theory (DFT) was employed. In situ AFM measurements revealed that TA exhibits specificity for binding to steps that advance in the [001] direction on the (100) surface of COM crystals. In the presence of TA, the hillocks on the (100) surface became rounded, the [001] step roughened, and inter‐step distances decreased (**Figure** **3**A; Movie S1, Supporting Information). The step velocity in the [001] direction on the (100) surface decreased monotonically with increasing TA concentration, becoming negligible above 0.5 mm (Figure 3I). The weakest inhibitor of COM crystallization identified in microfluidic assays, MA, was found to influence layer growth on the (100) surface. AFM measurements showed that the [001] step velocity and inter‐step distances decreased with increasing MA concentration, albeit to a lesser extent compared to TA (Figure 3B,J; Figure S16 and Movie S2, Supporting Information). Step roughening was also observed in the presence of MA. In contrast, MMA had little effect on layered growth (Figure S17, Supporting Information), with only a slight reduction in the rate of step advancement on the (100) surface (Figure 3K). According to theoretical models of spiral growth, the rate of crystal growth *R*_[100]_ perpendicular to the imaging surface is proportional to the step velocity *v*_[001]_.^[^ ^10b^ ^]^ The observed trends in [001] step growth inhibition are consistent with the results from the microfluidic assay, indicating that TA exhibits the strongest inhibition effect, followed by MA and MMA (Figure 3L; Figure S18, Supporting Information).

Continuous imaging of the COM (010) surface revealed that TA also inhibited the growth of hillocks bounded by (12$\bar{1}$) and (021) steps. At low TA concentration, the initially rectangular growth hillocks transformed into square shapes due to a seemingly preferred inhibition of (021) steps (Figure 3C; Figure S19, Supporting Information). When 1.0 mm TA was introduced, the step edges became serrated within 10 min of exposure to the solution. Notably, (12$\bar{1}$) steps located away from the nucleation center became significantly coarsened with small protrusions, and their advancement almost ceased (Figure 3D; Figures S19 and S20 and Movie S3, Supporting Information). New steps, however, continued to be produced from the nucleation site and advanced by spiral growth (Figure S21 and Movie S4, Supporting Information). Therefore, we postulate the continuous vertical advancement of layers by spiral growth, combined with the inhibition of their lateral advancement by growth inhibition, leads to the formation of protrusions on (010) surfaces at high TA concentrations. Indeed, this is reflected in the features observed in electron micrographs (Figure 3E; Figure S14, Supporting Information) and the proposed mechanism illustrated in Figure 3F (see also Figure S22, Supporting Information). Moreover, decreasing vertical advancement of layers on (010) surfaces with increasing TA concentration (Figure S21, Supporting Information) is in agreement with the decline in the growth rate of macroscopic protrusions observed in the microfluidic channels (Figure S13, Supporting Information). In contrast, for MA and MMA, their effects on COM (010) growth are relatively weak. Regarding CA, previous investigations suggest that it has a minimal effect on the growth of COM (010) surfaces.^[^ ^7^, ^10^ ^]^ This explains why (010) surfaces remained smooth in the presence of a high concentration of CA, as observed in the microfluidic assays. However, owing to the challenge of preparing COM crystals for AFM with flat and large apical (001), {021}, or {12$\bar{1}$} faces, the impact of TA on these surfaces remains elusive.

There are two common kinetic mechanisms regarding step inhibition: step pinning and kink blocking.^[^ ^6^, ^7^, ^16^ ^]^ Step pinning occurs when inhibitors adsorb onto terraces, acting as stoppers that obstruct the advancement of steps across the crystal surface. These adsorbed molecules induce step curvature, increasing the chemical potential of the curved steps and reducing the driving force for step growth. If the separation between a pair of adsorbed inhibitors Δ*x* is less than the diameter of the critical layer nucleus 2*r*_c_ at which the advancing step is undersaturated, the growth of existing steps is arrested (Figure 3G).^[^ ^17^ ^]^ On the contrary, kink blocking involves inhibitors binding to kink sites on steps, impeding the incorporation of growth units (Figure 3H). Since kinks are continuously generated at the step edge due to thermal fluctuations, kink blocking usually does not lead to a complete cessation of growth, even at high inhibitor concentrations.^[^ ^6^, ^18^ ^]^ In reality, inhibitors can bind to both kink and terrace sites, exerting a dual mode of action.^[^ ^7^ ^]^

The appearance of protrusions on [001] steps in the presence of TA and MA suggests a step‐pinning mechanism (Figure 3A,B); however, the immediate decrease of *v*_[001]_ with increasing inhibitor concentration (Figure 3I,J) and the approximately linear correlation between *v*_0_(*v*_0_‐*v*)^−1^ and *C*_inhibitor_^−1^ (Figure S23, Supporting Information) are characteristics of kink blocking.^[^ ^6^, ^7^ ^]^ Overall, these findings suggest that inhibitors TA and MA suppress [001] step movement through the cooperative action of step pinning and kink blocking. Given that increasing concentrations of inhibitors TA and MA lead to nearly complete arrest of step advancement, the step pinning mechanism dominates the inhibition of [001] layer growth. The presence of TA also results in a serrated appearance and reduced advancement of (021) and (12$\bar{1}$) steps on COM (010) surfaces; however, the relatively weak effect suggests that kink blocking is the dominant mechanism for inhibiting step advancement. A similar mechanism is observed for MA and MMA, which are less effective (i.e., weak inhibitors of step growth) and do not alter COM crystal morphology compared to the control.

To rationalize the observed experimental trends, we used density functional theory (DFT) calculations to assess inhibitor‐crystal interactions. The binding energies of deprotonated TA analogs docking to a [001] step on the (100) plane, as well as (021) and (12$\bar{1}$) steps on the (010) plane, were calculated with energy minimization (Figure 3M–O; Figure S25, Supporting Information). In the case of TA binding to a [001] acute step, the ─COO^−^ groups in TA bind to three Ca^2+^ sites, and its ‐OH group forms a hydrogen bond with one of the oxygen atoms of an oxalate ion in the COM crystal step riser (Figure 3M). Calculations of MA and MMA reveal only three ─COO^−^─Ca^2+^ bonds at the acute step (Figure 3O; Figure S25, Supporting Information). The calculated binding energy for TA at a [001] acute step is much higher (−173.6 kJ mol^−1^) than those of MA (−145.6 kJ mol^−1^) and MMA (−115.0 kJ mol^−1^), which indicates that TA has a much stronger affinity for [001] acute steps. This finding is consistent with AFM observations that TA is a more potent inhibitor of [001] step velocity compared to MA and MMA. DFT calculations also predict strong binding between TA and the (021) step on the (010) surface (−170.6 kJ mol^−1^) (Figure 3N), thus offering an explanation for its ability to reduce the (021) step velocity.

### 2.4 In Vitro Assays of COM Crystallization in Human Urine

**Figure 4:**
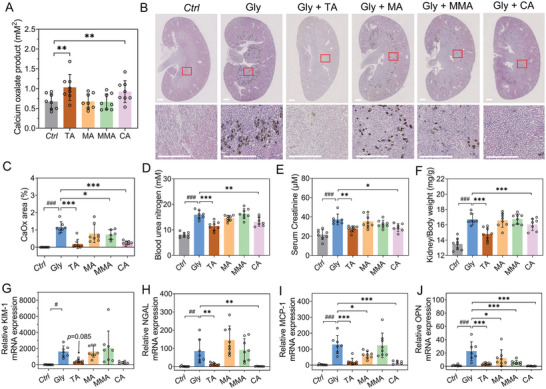
Assessing growth inhibitors in physiological environments. A) ULM assays in human urine expressed as the calcium oxalate (CaOx) concentration product in the presence of growth inhibitors (*C*_TA_ = *C*_MA_ = *C*_MMA_ = *C*_CA_ = 2 mm) compared to that of the control (*n* = 8 subjects). B) Representative Von Kossa staining images and the partially magnified photos of kidney sections. Dark areas indicate CaO_x_ crystals. All scale bars equal 500 µm. C) Quantification of crystal deposition in the Von Kossa‐positive area of kidney sections. D,E) Comparison of the (D) plasma blood urea nitrogen BUN and (E) serum creatinine levels. F) Ratio of the kidney weight to body weight. G–J) Quantitative representation of mRNA expression of (G) *KIM‐1*, (H) *NGAL*, (I) *MCP‐1*, and (J) *OPN* of mice kidney sections. Values are mean ± SD and *p* values obtained from ordinary one‐way ANOVA test. *n* = 7 Gly and CA groups, *n* = 8 *Ctrl*, TA, MA, and MMA groups in panels C–F, H, and J. *n* = 7 Gly, *n* = 6 CA groups, *n* = 8 *Ctrl*, TA, MA, and MMA groups in panels G and I. Two‐tailed one‐way ANOVA were used. (###) *p* < 0.001 as compared with *Ctrl* group. (*) *p* < 0.05, (**) *p* < 0.01, and (***) *p* < 0.001 as compared with Gly group. Source data are provided in the Source Data File.

To evaluate the effect of TA and its analogs on COM crystallization within a physiologically relevant environment, human urine from eight kidney stone patients was collected. The upper limit of metastability (ULM) of CaOx in urine samples was determined using a method detailed by Asplin and coworkers, which assesses the activity products of solutes in urine (see Experimental Section for details).^[^ ^6^, ^19^ ^]^ The ULM indicates the minimum oxalate ion concentration required to initiate the nucleation of COM in human urine. As shown in does not to be bold. **Figure** **4**A, compared to the untreated urine control, the ULM increased in the presence of TA. The rise in ULM induced by TA was also greater than that observed with CA, despite the latter possessing a more robust complexing ability and exhibiting a comparable inhibition effect on crystal growth. This indicates a strong inhibitory effect of TA on COM crystallization within the urinary milieu. Most discovered endogenous COM inhibitors, such as osteopontin, albumin, chondroitin sulfate, peptides, and citrate, preferentially influence the growth of the (100) surface.^[^ ^6^, ^7^, ^10^ ^]^ Thus, it is reasonable to expect that the apical‐face‐preferred inhibitor TA would have a synergistic effect on the COM crystallization with these endogenous inhibitors by virtue of their interactions with different surfaces, thereby demonstrating a stronger inhibition effect (illustration in Figure S26, Supporting Information).^[^ ^20^ ^]^ In contrast, the inhibitors MA and MMA had little impact on the ULM of CaOx (Figure 4A).

### 2.5 Inhibitors’ Effect on Renal CaOx Deposition and Associated Kidney Injury

We established a murine model of glyoxylate‐induced kidney CaOx nephrocalcinosis to assess the influence of TA on the in vivo deposition of CaOx crystals within the kidneys.^[^ ^21^ ^]^ Von Kossa staining was employed to examine the extent of CaOx crystal deposition in the murine kidneys. As shown in Figure 4B,C, TA dramatically reduced the crystal deposits within the kidney sections. Quantitative Polymerase Chain Reaction (PCR) analysis further revealed that the mRNA expression levels of *KIM‐1* and *NGAL*, recognized as typical markers of tubular injury, were lower in TA‐treated mice in comparison to their non‐treated counterparts (Figure 4G,H).^[^ ^22^ ^]^ This suggests that TA treatment reduces stone‐associated epithelial cell injury. A significant decrease in the mRNA levels of the inflammatory cytokine *MCP‐1* was also observed (Figure 4I), suggesting the attenuation of sterile inflammation at the lesion. Additionally, TA treatment effectively suppressed the glyoxylate‐induced up‐regulation of renal osteopontin (*OPN*) expression (Figure 4J), pointing to a mitigation of renal damage.^[^ ^23^ ^]^ When compared to mice afflicted with nephrocalcinosis, TA‐treated mice displayed decreased levels of plasma BUN, serum creatinine, and a reduced kidney/body weight ratio (Figure 4D–F), which collectively suggest improved excretory kidney function. Taken together, the results from the in vivo study employing a mouse model identify TA as a promising therapeutic to significantly attenuate the deposition of CaOx crystals and confer protective effects against kidney injury. It is noteworthy that the dose used in the in vivo experiments is relatively high, primarily for initial pharmacological studies aimed at evaluating its prevention of CaOx nephrocalcinosis. Further exploration of dose dependence and alternative administration routes, particularly oral administration, are crucial, as they may offer greater feasibility for the clinical usage of TA.

## 3 Discussion

CaOx crystal deposits within the kidneys pose a substantial risk to human health, potentially leading to a diverse range of kidney disorders through various tissue injuries.^[^ ^4^ ^]^ Unfortunately, the treatment options for CaOx nephrolithiasis have remained extremely limited. Currently, citrate is the primary treatment for patients afflicted by nephrocalcinosis; however, it comes with the drawback of gastrointestinal side effects that include (but are not limited to) upset stomachs, abdominal pain, and diarrhea.^[^ ^5^ ^]^ In this study, we introduce a promising alternative to citrate for preventing CaOx kidney stones: tartronic acid (TA). This molecule exhibits potent inhibition of COM crystallization in vitro and in vivo, with the potential to suppress CaOx‐induced renal injury.

Previous studies have emphasized the significance of anionic inhibitors in inhibiting COM growth, highlighting the role of negative charges in driving the interactions between inhibitors and Ca^2+^ ions on the crystal surface, thereby retarding crystal growth.^[^ ^7^, ^8^, ^24^ ^]^ This insight has prompted the exploration of molecules with a high degree of negative charge as potential inhibitors of COM crystallization.^[^ ^8c^ ^]^ To our knowledge, TA (possessing two acid groups) is the simplest polyprotic acid inhibitor reported for COM crystallization, and surprisingly, it surpasses citric acid (possessing three acid groups), a well‐established treatment for kidney stones, in inhibiting COM crystallization.^[^ ^5b^ ^]^ Our molecular simulations reveal that TA binds to COM surfaces by forming _(TA)_COO^−^…Ca^2+^_(COM)_…^−^COO_(TA)_ bonds with Ca^2+^ sites, and its hydroxyl group simultaneously engages in a hydrogen bond with the oxygen atoms of an oxalate ion in the COM crystal. It has been demonstrated that the number and placement of hydroxyl groups in the inhibitor play a critical role in the interaction with COM surfaces and the subsequent inhibitory performance.^[^ ^6^, ^7^, ^8^, ^10^, ^11^ ^]^ These hydroxyl groups significantly contribute to stabilizing the structure of the adsorbed molecule and facilitate hydrogen bonds with the crystal surface.

Many reported inhibitors of COM crystallization bind to basal {100} surfaces, which are the largest faces and possess the highest Ca^2+^ surface density (0.0542 Ca^2+^/Å^2^).^[^ ^6^, ^8^, ^10^ ^]^ In contrast, we observed that TA preferentially influences the growth rate in the [001] direction, which is the fastest direction of COM crystallization; however, the challenge of preparing COM crystals with flat and large apical surfaces for AFM measurements prevents us from further investigating the impact of TA on the (001), {021}, or {12$\bar{1}$} surfaces. We believe that TA's binding specificity underlies its potent inhibitory effect on COM crystallization, similar to previously reported modifiers (e.g., hydroxycitrate) that exhibit an affinity for targeting rapidly growing crystal surfaces.^[^ ^6^, ^10^, ^25^ ^]^ Anologies can be made to modifiers of ice, where the association between modifier binding to the surface of ice crystals and the inhibitory performance of antifreeze proteins has been reported.^[^ ^26^ ^]^ Moreover, TA demonstrates a pronounced affinity for the (010) surface, exerting a significant influence on the spiral growth and leading to a substantial roughness on this surface at elevated concentrations. In contrast, CA exhibits only marginal influence on the layer growth of the (010) surface.^[^ ^7^, ^10^ ^]^

Notably, TA exhibits renal excretion without undergoing a significant metabolism, irrespective of oral or injectable administration, with over half excreted in urine within 24 h post‐injection.^[^ ^14^ ^]^ This property is essential for the in vivo effectiveness of a crystallization inhibitor, as the interaction between the inhibitor and crystal surface significantly depends on preservation of the inhibitor's original structure. Urine composition is complex, containing a diverse range of molecules that influence the development of kidney stones and may interfere with crystallization inhibitors designed to prevent stone formation.^[^ ^16^, ^27^ ^]^ Therefore, the demonstration of TA efficacy in urine samples, especially within the context of kidney stone patients, is crucial for its in vivo application. Most identified endogenous inhibitors of COM preferentially affect the growth of the (100) surface.^[^ ^6^, ^7^, ^10^ ^]^ The apical‐face‐preferred inhibitor TA may potentially exhibit a synergistic effect on COM crystallization with these endogenous inhibitors. This synergy arises through cooperative interactions with different surfaces, potentially minimizing the required drug dose to mitigate potential side effects.^[^ ^20^ ^]^ Previous reports on COM crystal growth inhibitors support the idea of synergy, emphasizing that the most synergistic effect occurs when compounds bind to complementary faces of the crystal.^[^ ^6^, ^20^ ^]^ Additionally, TA's limited complexing ability, as observed in vitro, suggests that it may have little impact on the free calcium levels in *vivo* which is an essential cofactor in blood coagulation.^[^ ^28^ ^]^ Moreover, beyond its efficacy as a COM crystallization inhibitor, as demonstrated in this study, TA has previously shown numerous other pharmacological actions, such as inhibiting the conversion of carbohydrates into fat and regulating bone resorption.^[^ ^3^, ^13^, ^29^ ^]^ Considering that stone formers are often concurrently affected by hypertension, obesity, and osteoporosis, TA treatment holds the potential for comprehensive therapeutic benefits against kidney stone disease. However, before considering TA for prospective clinical trial in kidney stone prevention, it is imperative to attain a comprehensive understanding of its pharmacokinetics in humans, including bioavailability, biological half‐life, and urinary excretion rate. In addition, establishing optimal dosing regimens and ensuring long‐term safety and tolerability are equally essential considerations.

## 4 Conclusion

In summary, our study unveils tartronic acid (TA) as a highly efficient inhibitor of calcium oxalate crystallization. Through synergistic techniques encompassing bulk crystallization experiments, microfluidic assays, in situ atomic force microscopy (AFM) measurements, and density functional theory (DFT) calculations, we confirmed site‐specific adsorption of modifiers on COM surfaces and their corresponding mechanisms of inhibition. Our study demonstrated that TA can bind to kink and terrace sites, acting as a dual‐mode inhibitor. A comparative analysis of TA analogs underscores the remarkable impact of subtle variations in molecular structure, such as the addition of a single alcohol group, on binding selectivity and inhibition efficacy. An unexpected outcome is identifying an effective COM growth inhibitor with fewer acid sites than citric acid, which highlights the crucial role of hydrogen bonding for inhibitor–crystal interactions. Moreover, our investigations demonstrated that in vitro assessment of TA efficacy translated to in vivo studies wherein TA suppresses kidney stone development and associated tissue injury in a mouse model of hyperoxaluria. Collectively, these findings suggest TA is a promising alternative for the prevention and treatment of kidney stones. Our study also reveals the feasibility of developing crystal growth inhibitors to prevent CaOx stones, potentially motivating the development of therapies based on inhibitor‐crystal interactions for diseases associated with pathological crystallization.

## 5 Experimental Section

### Materials

The following chemicals were used as reagents: calcium chloride dihydrate (CaCl_2_∙2H_2_O, ≥99%, Sigma Aldrich), sodium oxalate (Na_2_C_2_O_4_, >99%, Sigma Aldrich), sodium chloride (NaCl, ≥99%, Sigma Aldrich), tartronic acid (TA, 98%, Aladdin), malonic acid (MA, 99.5%, Aladdin), methylmalonic acid (MMA, >98%, Aladdin), citric acid (CA, 99.5%, Macklin), sodium hydrogen carbonate (NaHCO_3_, ≥96%, Nanjing Chemical Reagent Co., Ltd.), sodium hydroxide (NaOH, ≥96%, Xilong Scientific), and hydrochloric acid (HCl, 36–38%, Nanjing Chemical Reagent Co., Ltd.). Deionized (DI) water used in all experiments was purified through an RO‐DI laboratory water purification system (Spring‐R10, 18.2 MΩ). All reagents were used as received without further purification.

### Bulk Crystallization Assays

Batch crystallization was carried out following a documented protocol in a 20‐ml glass vial.^[^ ^6b^ ^]^ Initially, NaCl was dissolved in DI water to prepare a 150 mm solution. Then, CaCl_2_ and Na_2_C_2_O_4_ were dissolved in the NaCl solution to prepare stock solutions, each with a concentration of 10 mm. Next, NaCl (8.6 ml, 150 mm) solution was added in the glass vial, followed by the addition of CaCl_2_ (0.7 ml, 10 mm) stock solution. A clean glass slide (≈1.5 × 1.5 cm^2^) was placed at the bottom of the vial to collect the crystals for microscopy. The pH of the COM growth solution was adjusted to 6.2 ± 0.2 using an Orion 2‐Star Bentchtop pH meter with a ROSS Ultra electrode (8102BNUWP). The sample vial was then placed in an oven set to 60 °C for 1 h to ensure the solution reached the set point temperature for crystallization. Subsequently, Na_2_C_2_O_4_ (0.7 ml, 10 mm) stock solution was added to the vial dropwise while continuously stirring. To investigate the effect of growth inhibitors on COM crystallization, an appropriate quantity of the inhibitor was added to the growth solution before Na_2_C_2_O_4_ addition. The final growth solution had a composition of CaCl_2_ (0.7 mm), Na_2_C_2_O_4_ (0.7 mm), NaCl (150 mm), and inhibitor (*C*_inhibitor_ = 0–1.0 mm), and a total volume of 10 ml. Crystallization was performed at 60 °C for 72 h under static conditions. The glass slide was removed from the solution, gently washed with DI water, and dried at room temperature before analysis.

### Characterization of COM Crystal

The size and morphology of COM crystals prepared in the absence and presence of inhibitors were assessed by polarized optical microscopy using an Olympus BX53 instrument. At least ten images of representative areas were captured for each slide. The dimensions ([100] length and [010] width) of crystals were measured for a minimum of 158 crystals from three separate batches to obtain an average [001]/[100] aspect ratio. The COM [100] thickness was measured from electron micrographs (FEI, Quanta 250 FEG). Scanning electron microscope (SEM) samples were prepared by gently pressing COM crystals on the glass slide to carbon tape to transfer crystals to the sample disk. Each sample was coated with a layer of carbon (≈20 nm thick) to reduce the effects of electron beam charging. X‐ray diffraction patterns were conducted on the COM crystals grown on the glass slides, using a Rigaku SmartLab SE X‐ray diffractometer to examine the surface of COM crystals. Cu‐Kα X‐radiation (*λ* = 1.540593 Å) was employed at 40 kV and 40 mA, within the 2θ range of 5–55°, with a scan rate of 5° min^−1^.

### Kinetics of COM Crystallization

Kinetic studies of COM crystallization were performed by measuring the depletion of free Ca^2+^ ions using an Orion 9720BNWP ion plus calcium ion‐selective electrode (ISE). A growth solution (pH = 6.2±0.2) with a molar composition of CaCl_2_ (0.5 mm), Na_2_C_2_O_4_ (0.5 mm), NaCl (150 mm), and inhibitor (*C*_inhibitor_ = 0–8 mm) was prepared. Inhibitors were incorporated into the solution after the addition of CaCl_2_ and prior to the addition of Na_2_C_2_O_4_. Crystallization was initiated by the introduction of 0.4 mg COM seed crystals (0.4 mg), and ISE measurements were conducted under continuous stirring (600 rpm) in a conical flask immersed in a thermostatic water bath controlled at 37 °C with a volume of 100 ml. Plots of consumed calcium ion concentration as a function of time were generated for each inhibitor concentration. A mimimum of four measurements were conducted for each data point. The data were normalized by subtracting the concentration at the initial time point. The approximate linear slope of these curves, observed within the first 10 to 40 min of crystallization, was determined to represent the rate of Ca^2+^ depletion. The efficacy of growth inhibitors was determined using percent inhibition, which was calculated by comparing the change in slope of the growth curve in the presence of the inhibitor against that observed in the absence of the inhibitor. Prior to each ISE measurement, the electrode was calibrated with calcium standards prepared by first diluting a commercial calcium solution (0.1 m, Orion Ion Plus) in NaCl (150 mm) to three different concentrations (10^−2^,10^−3^, and 10^−4^ m Ca^2+^).

The complexation between inhibitors and Ca^2+^ was assessed by measuring the depletion of free Ca^2+^ ions in the presence of different concentrations of each inhibitor using calcium ISE. A solution (pH = 6.2 ± 0.2) with a molar composition of CaCl_2_ (0.5 mm) and NaCl (150 mm) was prepared. Inhibitors were gradually added to the solution, incrementally increasing to the desired concentration (0–8 mm). ISE measurements were carried out under continuous stirring at 600 rpm in a conical flask immersed in a thermostatic water bath controlled at 37 °C. Each data point was based on a minimum of three measurements. The complexation of growth inhibitors was described using the relative concentration of free Ca^2+^, determined by comparing the concentration of free Ca^2+^in the presence of the inhibitor against that observed in the absence of the inhibitor. Before each ISE measurement, the electrode was calibrated with calcium standards prepared by first diluting a commercial calcium solution (0.1 mol, Orion Ion Plus) in NaCl (150 mm) to three different concentrations (10^−2^,10^−3^, and 10^−4^ m Ca^2+^).

### Microfluidic Assays

A microfluidic device described in the Supporting Information File was employed for in situ crystallization studies based on a previously reported protocol.^[^ ^30^ ^]^ For testing at physiological temperatures, the microfluidic device was placed on a thermoplate (Tokai Hit, TPi‐TCSX) and the temperature of the device was held at 37 °C. The microfluidic channels were first flushed thoroughly with DI water. Then, the growth experiment was conducted in two steps: preparation of COM seeds and subsequent crystal growth in the absence and presence of different amounts of inhibitors. All solutions were delivered into the channels using a dual syringe pump (LangerPump, LSP02‐2B). Two different nucleation methods were used to generate crystals with (100) or (010) faces oriented upward in the microfluidic channels. In the first approach (Method 1), a solution containing CaCl_2_ (2.0 mm) and NaCl (150 mm) was mixed via a Y‐connector with a second solution containing Na_2_C_2_O_4_ (2.0 mm) and NaCl (150 mm). The mixed growth solution was introduced into PDMS chips for in situ preparation of COM seeds at a flow rate of 1 ml h^−1^ for 30 min. Seeds in the microfluidic channels with (100) faces oriented upward can be obtained by the first method. In the second approach (Method 2), a solution containing CaCl_2_ (2.24 mm) and NaCl (150 mm) was mixed via a Y‐connector with a second solution containing Na_2_C_2_O_4_ (2.24 mm), NaHCO_3_ (8.08 mm) and NaCl (150 mm). The mixed growth solution was introduced into the PDMS chips for crystal growth at a flow rate of 1 ml h^−1^ for 30 min. Seeds in the microfluidic channels with either (100) or (010) faces oriented upward could be obtained by the second method.

COM crystal size and morphology were determined in the microfluidic system using a NIKON Ts2R inverted optical microscope. For growth, two solution components were prepared in individual syringes. One solution contained CaCl_2_ (1.0 mm) and NaCl (150 mm) and the second solution contained Na_2_C_2_O_4_ (1.0 mm), NaCl (150 mm), and inhibitor (2*C*_inhibitor_, *C*_inhibitor_ = 0–2.0 mm). The two solutions were mixed through a Y‐connector, producing a final composition of Ca^2+^ (0.5 mm), Ox^2−^ (0.5 mm), NaCl (150 mm), and inhibitor (*C*_inhibitor_ mm). The fully mixed growth solution was introduced into seeded PDMS chips. Time‐resolved imaging of COM crystal growth was performed to quantify the kinetics of COM crystallization. Crystal size was analyzed from the images using ImageJ software (National Institutes of Health, Bethesda, MD, USA). Growth rate in the [001] and [010] directions of COM crystals were measured from a minimum of 10 crystals per trial and at least six individual trials. Growth rate in the [100] directions of COM crystals were measured from a minimum of 10 crystals per trial and at least two individual trials. From the change in crystal length over time, a growth rate *R* was determined for each experimental condition. The relative growth rate (RGR) was calculated as

(1) $$\mathrm{RGR}\;=\frac{R_{inhibitor}}{R_{control}}\;$$

where *R*_inhibitor_ and *R*_control_ represent growth rates in the presence and absence of inhibitors, respectively.

### Atomic Force Microscopy (AFM)

In situ AFM was performed using a Digital Instruments Multimode Nanoscope IV (Santa Barbara, CA) to examine topographical images of COM crystals and capture the dynamics of (100) and (010) surface growth in real‐time. COM crystals generated in bulk crystallization assays on a small piece of glass were mounted on an AFM specimen disk (Ted Pella) by double‐sided tapes in accordance with previously reported protocols.^[^ ^6b^ ^]^ AFM images were collected in contact mode using a Bruker ScanAsyst‐Fluid probe (silicon nitride, 0.7 N m^−1^ spring constant) with a scan rate of 3–7 Hz at 256 lines per scan. For in situ AFM measurements, a growth solution with a supersaturation ratio of *S* = 4.1 was prepared with a composition of CaCl_2_ (0.18 mm), Na_2_C_2_O_4_ (0.18 mm), and inhibitor (*C*_inhibitor_ = 0 – 2.0 mm). The AFM instrument was equipped with a fluid sample cell (model MTFML) containing two ports for inlet and outlet flow to maintain constant supersaturation during continuous imaging. The growth solution was delivered to the liquid cell using a dual syringe pump (CHEMYX, Fusion 200) with an in‐line mixing configuration to combine CaCl_2_ and Na_2_C_2_O_4_ solutions with a combined flow rate of 0.2 ml min^−1^. Inhibitors were introduced into the Na_2_C_2_O_4_ solution at the appropriate concentration. The velocity of step advancement and changes in hillock morphology on COM (010) and (100) surfaces were measured in the absence or presence of inhibitors. The reported step velocity was the average of at least 5 measurements of different steps.

### Molecular Modeling Using Density Functional Theory (DFT) Calculations

The binding interactions between TA analogs and the [001] step on the (100) face, the (021) step on the (010) face, and the (12$\bar{1}$) step on the (010) face were calculated by density functional theory (DFT) using the DMol3 module in the Materials Studio 2017 software. The calculations employed the gradient‐corrected functional of generalized gradient approximation (GGA) with PBE. The long‐range dispersion was considered on the basis of Grimme's correction (DFT‐D). The COSMO continuum solvation model was used to simulate the aqueous environment for the calculations (the solvent was water with dielectric constant *ε* = 78.54). Structure optimization was performed to obtain the minimum energy configurations.

The whewellite (monoclinic) crystal unit cell was used to build the COM crystals (with H_2_O molecules being removed and simulated by implicit solvent effects). There are two ways to build a [001] step based on the crystal structure, both of which have the same step base in the (100) plane but a different step riser. Both the [001] acute and abuse steps on the (100) face consisted of 252 atoms (Ca_36_C_72_O_144_). The (021) and (12$\bar{1}$) steps on the (010) plane consisted of 224 (Ca_32_C_64_O_128_) and 175 atoms (Ca_25_C_50_O_100_), respectively. The [001] step on the (100) plane, and the (021) and (12$\bar{1}$) steps on the (010) plane were kept frozen, and the inhibitors were allowed to fully relax in the calculations. The binding energy of inhibitors to crystal surfaces was defined as

(2) $$\mathrm{Bind}\mathrm{ing}\;\mathrm{Energy}\;=E_{\mathrm{inhi}\mathrm{bitor}+\mathrm{COM}}\;-E_{\mathrm{inhi}\mathrm{bitor}}-E_{\mathrm{COM}}$$

where *E*_x_ represents the total electronic energy of species *x*. For calculations of “inhibitor + COM” species, the deprotonated forms of the acids were placed on the COM surfaces and sodium ions (Na^+^) were placed at a position far away from the interaction center to neutralize the system. The energy of the inhibitor in the gas phase, labeled as *E*_inhibitor_, corresponds to molecules in their protonated (neutral) state. The pH of the crystallization media impacts the net charge of the inhibitor. The inhibitors selected for this study (TA, MA, and MMA) had two dissociation constants corresponding to each of their carboxylic acid groups.

### In Vitro Assays of COM Crystallization in Human Urine

Urine aliquots were obtained over a 24 h urine collection period from eight patients with kidney stones provided by Affiliated Drum Tower Hospital, Medical School of Nanjing University (Nanjing, China). All samples were collected and analyzed with informed consent. Before introducing inhibitors (TA, CA, MA, and MMA) (2.0 mm) and adjusting the pH to 6.0, human urine aliquots were first centrifuged and filtered using a 0.45 µm syringe filter to remove debris. The concentrations of Ca^2+^ and Ox^2−^ in human urine samples were determined by inductively coupled plasma mass spectrometry (ICP‐MS) (ThermoFisher, ICAP QC) and the oxalate assay kit (Sigma‐Aldrich, MAK179) following the manufacturer's instructions, respectively. A 200 µl portion of the pretreated urine aliquots was added to 60 wells of the 96‐well microplates for different urine groups. Solutions with Ox^2−^ concentrations of 10, 15, 20, and 25 mm were prepared by dissolving Na_2_C_2_O_4_ in NaCl (150 mm) solution. The pH of each solution was adjusted to 6.0. To initiate CaOx precipitation, the prepared Ox^2−^ solution of increasing concentration was introduced into each well, with a volume not exceeding 20 µl. The microplates were then transferred to a shaker for 3 h at 37 °C and 100 rpm. The turbidity of solutions in each well was measured at 620 nm wavelength using a multifunctional ELISA microplate reader (POLARstar Omega). The point of CaOx crystallization was determined by the sudden increase in turbidity, in which the total oxalate concentration comprised the added amount and the initial amount in the urine. The product of the calcium oxalate concentration was used to present the upper limit of metastability. Experimental groups of each human urine sample were repeated at least three times, and the results were averaged.

### In Vivo Studies

All the animal experiments were performed following the Guidelines for Care and Use of Laboratory Animals of Nanjing University and approved by the Animal Ethics Committee of the College of Life Sciences, Nanjing University, China. Eight‐week‐old male C57BL/6J mice were used for this study and purchased from Gempharmatech Co., Ltd. (Nanjing, China). All animals were kept in separate cages (temperature, 25 ± 1 °C; humidity, 50%) and fed on a healthy diet. Animals were randomly divided into six groups (*n *= 7–8): the normal control (*Ctrl*), glyoxylate‐induced nephrolithiasis (Gly), the positive control (glyoxylate‐induced nephrolithiasis plus citric acid, CA), and three treatment groups, including glyoxylate‐induced nephrolithiasis plus tartronic acid (TA), glyoxylate‐induced nephrolithiasis plus malonic acid (MA), glyoxylate‐induced nephrolithiasis plus methylmalonic acid (MMA). The nephrolithiasis mouse models were established via intraperitoneal injection of 60 mg kg^−1^ d^−1^ glyoxylate for 14 days, and mice in the normal control group were injected with the same volume of saline. Concurrently, mice in the treatment groups received 1.56 mm kg^−1^ crystal growth inhibitors (TA 187.5, MA 162.5, and MMA 118.09 mg kg^−1^) twice a day, and mice in the positive control group received 300 mg k^−1^g (1.56 mm kg^−1^) CA twice a day via intraperitoneal injection. The dose of CA in this study was determined based on the clinical dose of potassium citrate, maintaining consistent molar concentration ratios with TA, MA, and MMA with CA.^[^ ^31^ ^]^ Further details are provided in the Supplementary Experimental Section. On day 14, the blood of mice was drawn under pentobarbital anesthesia from the orbital venous plexus. Serum levels of BUN and creatinine (Shanghai Yuanye Bio‐Technology Co., Ltd) were determined (Beijing Solarbio Science & Technology Co., Ltd). All mice were sacrificed by cervical dislocation at the end of the study. The kidneys were extracted to examine CaOx stone formation and RNA preparation.

In vivo crystal deposition in extracted mouse kidneys was examined using von Kossa staining (Wuhan Servicebio Technology Co., Ltd.) and the stained slices were visualized under a slice scanner (NanoZoomer 2.0 RS, Hamamatsu) to determine crystal deposition. Crystal formation, calculated as the percent area of CaOx crystal deposition per kidney section, was assessed using ImageJ software (National Institutes of Health, Bethesda, MD, USA).

Total RNA was extracted from mouse kidney sections and reverse transcribed to acquire cDNA using the TRIzol reagent (Invitrogen, Carlsbad, CA, USA) and the PrimeScript RT reagent kit (TaKaRa, Shiga, Japan), followed by Polymerase Chain Reaction (PCR) amplification using SYBR Green master mix (Yeasen, Shanghai, China) according to a standard protocol. The mRNA expression levels of Kidney Injury Molecule‐1 (*KIM‐1*), Neutrophil gelatinase‐associated lipocalin (NGAL), Human macrophage chemoattractant protein‐1 (*MCP‐1*), and osteopontin (*OPN*) were quantified by real‐time RT‐PCR using the StepOnePlus with GAPDH as the reference gene. Primer sequences are listed in Table S1 (Supporting Information). Relative mRNA expression was calculated using the 2−^ΔΔCT^ method.^[^ ^32^ ^]^

### Statistical Analysis

Statistical analysis and graphs were prepared using GraphPad Prism 8.0 (La Jolla, CA, USA). Experimental data were presented as the mean ± standard deviation. Two‐tailed Student's T‐test and ordinary one‐way ANOVA testing followed by post‐hoc Dunnett's multiple comparison was used to determine the *p* value. The statistical significance threshold was set at *p* < 0.05. *p ≤ 0.05, **p ≤ 0.01, ***p ≤ 0.001.

## Conflict of Interest

The authors declare no conflict of interest.

## Author Contributions

Y.S., S.L., and X.L. contributed equally to this work. Y.S. and T.C. conceived and planned the study. Y.S. carried out bulk crystal growth studies, kinetics of COM crystallization, microfluidic assays, and theoretical modeling. X.L. and W.Y. planned and carried out in vivo studied. S.L. and V.P.C. carried out in situ AFM measurements. J.‐Y.Z. performed microfluidic assays and completed in vitro assays of COM crystallization in human urine. L.M., C.‐M.L., and Y.‐F.R. performed microfluidic assays. Y.‐H.S. carried out kinetics of COM crystallization. Y.S., S.L., J.D.R., and T.C. analyzed the experimental data and wrote the manuscript. All the authors discussed the results and approved the final version of the manuscript.

## Supporting information

Supporting Information

Supplemental Movie 1

Supplemental Movie 2

Supplemental Movie 3

Supplemental Movie 4

## Data Availability

The data that support the findings of this study are available from the corresponding author upon reasonable request.

## References

[1] a) E. M. Worcester , F. L. Coe , N. Engl. J. Med. 2010, 363, 954; 20818905 10.1056/NEJMcp1001011 PMC3192488

[2] a) O. W. Moe , Lancet 2006, 367, 333; 16443041 10.1016/S0140-6736(06)68071-9

[3] a) S. R. Khan , B. K. Canales , P. R. Dominguez‐Gutierrez , Nat. Rev. Nephrol. 2021, 17, 417; 33514941 10.1038/s41581-020-00392-1

[4] a) S. R. Mulay , H.‐J. Anders , Nat. Rev. Nephrol. 2017, 13, 226; 28218266 10.1038/nrneph.2017.10

[5] a) N. L. Miller , J. E. Lingeman , Nat. Clin. Pract. Urol. 2006, 3, 236; 16691216 10.1038/ncpuro0480

[6] a) J. D. Rimer , Z. An , Z. Zhu , M. H. Lee , D. S. Goldfarb , J. A. Wesson , M. D. Ward , Science 2010, 330, 337; 20947757 10.1126/science.1191968 PMC5166609

[7] a) W. Ma , V. A. Balta , W. Pan , J. D. Rimer , D. J. Sullivan , P. G. Vekilov , Commun. Biol. 2023, 6, 783; 37500754 10.1038/s42003-023-05046-z PMC10374632

[8] a) J. Chung , R. Sosa , J. D. Rimer , Cryst. Growth Des. 2017, 17, 4280;

[9] C. A. Dauw , Y. Yi , M. J. Bierlein , P. Yan , A. F. Alruwaily , K. R. Ghani , J. S. Wolf Jr. , B. K. Hollenbeck , J. M. Hollingsworth , Urology 2016, 93, 45. 27041472 10.1016/j.urology.2016.03.030

[10] a) S. R. Qiu , A. Wierzbicki , E. A. Salter , S. Zepeda , C. A. Orme , J. R. Hoyer , G. H. Nancollas , A. M. Cody , J. J. D. Yoreo , J. Am. Chem. Soc. 2005, 127, 9036; 15969581 10.1021/ja043591s

[11] L. Wang , X. Guan , R. Tang , J. R. Hoyer , A. Wierzbicki , J. J. D. Yoreo , G. H. Nancollas , J. Phys. Chem. B 2008, 112, 9151. 18611047 10.1021/jp804282u PMC2743538

[12] D. B. Hauzea , K. L. Keesa , C. W. Manna , H. F. III , R. Murrills , J. Matteo , F. Bex , B. Bhat , V. Coleburn , Lett. Drug Des. Discovery 2005, 2, 201.

[13] a) C. Granchi , I. Paterni , R. Rani , F. Minutolo , Future Med. Chem. 2013, 5, 1967. 24175747 10.4155/fmc.13.151 PMC3952072

[14] a) L. G. Wesson , Nature 1961, 189, 147;13731460

[15] A. Millan , Cryst. Growth Des. 2001, 1, 245.

[16] B. G. Alamani , J. D. Rimer , Curr. Opin. Nephrol. Hypertens. 2017, 26, 256. 28410252 10.1097/MNH.0000000000000330

[17] A. G. Shtukenberg , M. D. Ward , B. Kahr , J. Cryst. Growth 2022, 597, 126839.

[18] P. G. Vekilov , Cryst. Growth Des. 2007, 7, 2796.

[19] J. R. Asplin , J. H. Parks , M. S. Chen , J. C. Lieske , F. G. Toback , S. N. Pillay , Y. Nakagawa , F. L. Coe , Kidney Int. 1999, 56, 1505. 10504502 10.1046/j.1523-1755.1999.00682.x

[20] T. Berger , K. Meister , A. L. DeVries , R. Eves , P. L. Davies , R. Drori , J. Am. Chem. Soc. 2019, 141, 19144. 31710222 10.1021/jacs.9b10905

[21] A. Okada , S. Nomura , Y. Higashibata , M. Hirose , B. Gao , M. Yoshimura , Y. Itoh , T. Yasui , K. Tozawa , K. Kohri , Urol. Res. 2007, 35, 89. 17393196 10.1007/s00240-007-0082-8

[22] J. H. Ix , M. G. Shlipak , Am. J. Kidney Dis. 2021, 78, 719. 34051308 10.1053/j.ajkd.2021.03.026 PMC8545710

[23] S. R. Khan , J. M. Johnson , A. B. Peck , J. G. Cornelius , P. A. Glenton , J. Urol. 2002, 168, 1173. 12187263 10.1016/S0022-5347(05)64621-6

[24] L. Wang , S. R. Qiu , W. Zachowicz , X. Guan , J. J. D. Yoreo , G. H. Nancollas , J. R. Hoyer , Langmuir 2006, 22, 7279. 16893227 10.1021/la060897z

[25] D. Kim , J. D. Rimer , J. R. Asplin , Urolithiasis 2019, 47, 311. 30915494 10.1007/s00240-019-01125-1

[26] a) L. L. Olijve , K. Meister , A. L. DeVries , J. G. Duman , S. Guo , H. J. Bakker , I. K. Voets , Proc. Natl. Acad. Sci. USA 2016, 113, 3740; 26936953 10.1073/pnas.1524109113 PMC4833260

[27] a) A. Marimuthu , R. N. O'Meally , R. Chaerkady , Y. Subbannayya , V. Nanjappa , P. Kumar , D. S. Kelkar , S. M. Pinto , R. Sharma , S. Renuse , R. Goel , R. Christopher , B. Delanghe , R. N. Cole , H. C. Harsha , A. Pandey , J. Proteome Res. 2011, 10, 2734; 21500864 10.1021/pr2003038 PMC4213861

[28] G. Lee , G. M. Arepally , J. Clin. Apher. 2012, 27, 117. 22532037 10.1002/jca.21222 PMC3366026

[29] G. Caselli , M. Mantovanini , C. A. Gandolfi , M. Allegretti , S. Fiorentino , L. Pellegrini , G. Melillo , R. Bertini , W. Sabbatini , R. Anacardio , G. Clavenna , G. Sciortino , A. Teti , J. Bone Miner. Res. 1997, 12, 972. 9169358 10.1359/jbmr.1997.12.6.972

[30] a) R. D. Sosa , X. Geng , M. A. Reynolds , J. D. Rimer , J. C. Conrad , Lab Chip 2019, 19, 1534; 30951060 10.1039/c9lc00061e

[31] a) R. Phillips , V. S. Hanchanale , A. Myatt , B. Somani , G. Nabi , C. S. Biyani , Cochrane Database Syst. Rev. 2015, 2015, CD010057; 26439475 10.1002/14651858.CD010057.pub2 PMC9578669

[32] K. J. Livak , T. D. Schmittgen , Methods 2001, 25, 402. 11846609 10.1006/meth.2001.1262

