# Supplementary material for: Tartronic Acid as a Potential Inhibitor of Pathological Calcium Oxalate Crystallization
Source: Adv Sci (Weinh). 2024 Apr 22;11(21):2400642. doi: 10.1002/advs.202400642 (PMC11151019; doi:10.1002/advs.202400642)
Supplement: Supplementary file 1 — Supporting Information [file ADVS-11-2400642-s005.pdf]

ADVANCED SCIENCE
Open Access

Supporting Information

for *Adv. Sci.*, DOI 10.1002/advs.202400642

Tartronic Acid as a Potential Inhibitor of Pathological Calcium Oxalate Crystallization

Yuan Su, Si Li, Xin Li, Jing-Ying Zhou, Vraj P. Chauhan, Meng Li, Ya-Hui Su, Chun-Mei Liu, Yi-Fei Ren, Wu Yin, Jeffrey D. Rimer* and Ting Cai**

Supporting Information

Tartronic acid as a Potential Inhibitor of Pathological Calcium Oxalate Crystallization

Yuan Su[1,2], Si Li[3], Xin Li[4], Jing-Ying Zhou[2], Vraj P. Chauhan[3], Meng Li[2], Ya-Hui Su[2], Chun-Mei Liu[2], Yi-Fei Ren[2], Wu Yin[4], Jeffrey D. Rimer[3*] and Ting Cai[1,2*]*

Supplementary Experimental Section

Preparation of crystal seeds for kinetics study of COM crystallization: COM seeds were prepared by the dropwise addition of $CaCl_2$ (0.2 M, 500 ml) and $Na_2C_2O_4$ (0.2 M, 500 ml) into DI water (500 ml) at 70°C. The suspension was stirred continuously at this temperature for 9 hours to allow for complete conversion of the precipitate to COM. The solid was then filtered and washed with DI water until free from residual chloride ions. The dried solid was ground and sieved through 200-mesh sieves before using.

Fabrication of microfluidic devices: The microchannel design was drafted using AutoCAD software (Autodesk) and fabricated using standard photolithography and polymer casting techniques. A negative photoresist with 100 µm thick features was patterned on a 4-inch silicon wafer using photolithography. Subsequently, a mixture of polydimethylsiloxane (PDMS) prepolymer and curing agent (volume ratio of 10:1) was degassed for 30 minutes and poured over the microchannel molds to a thickness of 3 mm. PDMS molds were cured at 80°C for 30 minutes, after which devices were extracted using a razor blade. Inlet and outlet ports were created using a 1.6 mm biopsy punch. Finally, PDMS molds were bound onto the glass substrates after plasma activation.

Supersaturation calculation: The driving force behind the crystal growth of COM in supersaturated solutions is the change in Gibbs free energy from the supersaturated to saturated state. In the manuscript, the supersaturation ratio (S) was calculated by

$$S = \left(\frac{a_{(Ca^{2+})}a_{(Ox^{2-})}}{K_{sp}}\right)^{0.5} \qquad (S1)$$

where $a_{(Ca^{2+})}$ and $a_{(Ox^{2-})}$ are the activities of calcium and oxalate ions, and K_{sp} is the solubility activity product. The solubility activity product K_{sp} of COM crystals is 2.20 × 10^{-9} mol^2 l^{-2} at 37°C and 1.45 × 10^{-9} mol^2 l^{-2} at 20°C.[1] We calculated the activity coefficients γ_i (i=Ca^{2+} or Ox^{2-}) using the Davies equation

$$\log\gamma_i = -0.5\, z_i^2\left(\frac{\sqrt{I}}{1+\sqrt{I}} - 0.3I\right) \quad (S2)$$

where z_i and I ($I = 0.5\sum z_i^2 X_i$) represent the ion valence and the ionic strength, respectively, and X_i is the concentration of "*i*th" species.

Step velocity calculation: The step advancement v of layers on surfaces of COM crystals is expected to vary according to the equation

$$v = \beta\omega\left[\left(a_{(Ca^{2+})}a_{(Ox^{2-})}\right)^{0.5} - \left(a_{e(Ca^{2+})}a_{e(Ox^{2-})}\right)^{0.5}\right] \quad (S3)$$

where $a_{e(Ca^{2+})}$ and $a_{e(Ox^{2-})}$ is the equilibrium activity, ω is the molecular volume in the crystal, and β is the kinetic coefficient.[2] Incorporating equation S1 into equation S3, we obtain

$$v = \beta\omega(S-1) \quad (S4)$$

Dose selection of crystal growth inhibitors in in vivo experiments: Allometric scaling is the most widely accepted approach for dose extrapolation based on normalization of dose-to-body surface area.[3] Human equivalent dose (HED) can be determined using the following equations:

1. Conversion dose based on surface area correction factors

HED (mg/kg) = Animal dose (mg/kg) × ($Weight_{animal}$ (kg) / $Weight_{human}$ (kg))$^{(1-b)}$ (*S*5)

where *b* is an allometric exponent for body surface area, typically set at 0.67.

2. Conversion based on a mg/kg to a mg/m^2 dose

HED (mg/kg) = Animal dose (mg/kg) × (Animal k_m/ Human k_m) (*S*6)

where k_m is a factor estimated by dividing the average body weight (kg) of the species by its body surface area (m^2).

In this study, citric acid (CA) dose for a mouse model (weighing approximately 20 g) is 300 mg/kg (equivalent to 1.56 mM/kg) twice a day. The calculation of HED for CA, using Equations S5 and S6, yields values of 45.67 mg/kg and 24.32 mg/kg. Thus, for a 60 kg human, the proposed doses are 2.7 g (potassium citrate 4.3 g) and 1.5 g (potassium citrate 2.4 g) twice per day for CA. These values align with the documented safe doses of potassium citrate, the primary medication for nephrolithiasis used in human study, as listed in the table below.[4] The dose of CA in this study was determined based on the clinical dose of potassium citrate, maintaining consistent molar concentration ratios with tartronic acid

(TA), malonic acid (MA), and methylmalonic acid (MMA) with CA.[4] It is noteworthy that potassium citrate is typically adminstrated orally in clinical settings. However, in our *in vivo* experiments, we employed intraperitoneal administration to assess the therapeutic effects of crystal growth inhibitors on calcium oxalate (CaOx) nephrocalcinosis. This route was selected for its practicality, safety for animals, and faster drug absorption, all of which are advantageous for initial proof-of-concept studies. Given the crucial role of administration route and its impact on bioavailability, further *in vivo* experiment are imperative to explore alternative routes of administration for TA, particularly oral administration, as they may offer greater feasibility for the clinical usage of TA.

The administration dose and duration of potassium citrate in reference.

	Dose / day	Period	Reference
Potassium citrate	60 mEq (6.4 g)	3 years	[4f]
Potassium citrate	60 mEq (6.4 g)	12 months	[4b]
Potassium citrate	40 mEq (4.32 g)	40.6 ± 31.1 months	[4c]
Potassium citrate	81 mEq (8.7 g)	12 months	[5]
Sodium-potassium citrate	6 - 8 g	12 months	[6]

Supplementary Figures

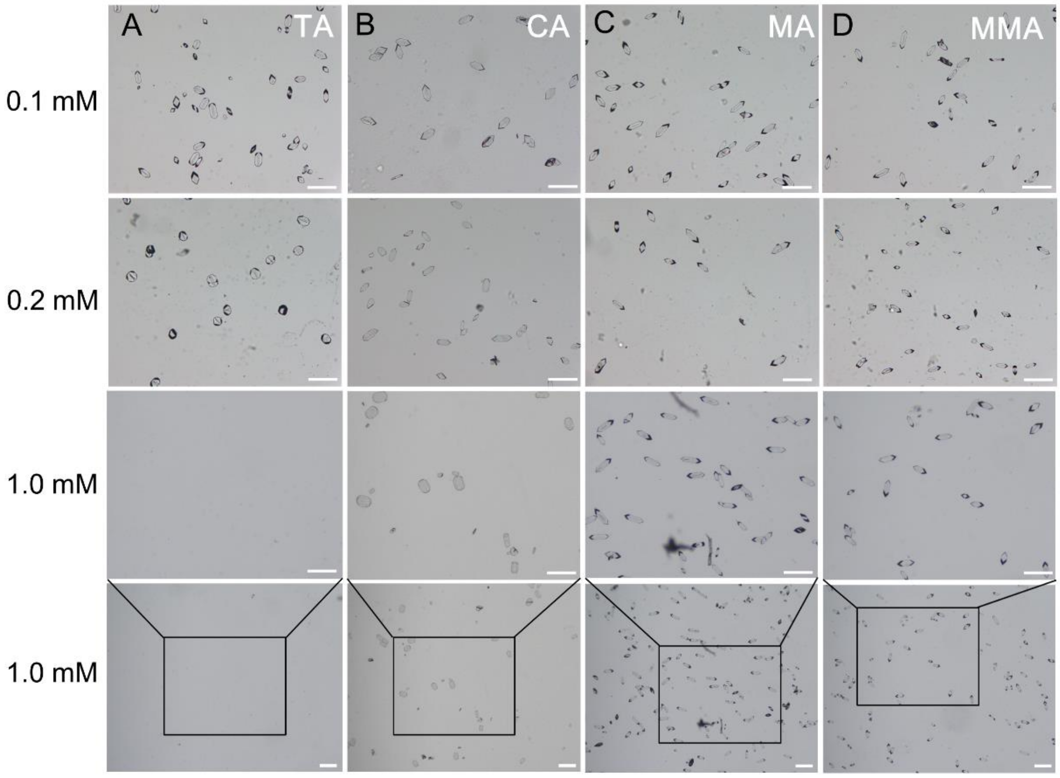

Figure S1. Optical micrographs of COM crystals prepared in the presence of (A) tartronic acid (TA), (B) citric acid (CA), (C) malonic acid (MA), and (D) methylmalonic acid (MMA). Scale bars equal 200

μm. In the presence of 0.1 mM TA, the apical tips of the COM crystals became blunted. At a TA concentration of 0.2 mM, the COM (010) surface disappeared, and further increasing to 1.0 mM resulted in the complete inhibition of COM crystallization. In contrast, the morphology of COM crystals remained constant in the presence of different concentrations of MA and MMA. Introduction of the well-established inhibitor, CA, led to a significant thinning of COM crystals with blunt apical tips. Despite a low crystal number density, COM continues to crystallize in the presence of 1.0 mM CA, indicating that CA is a less effective inhibitor than TA.

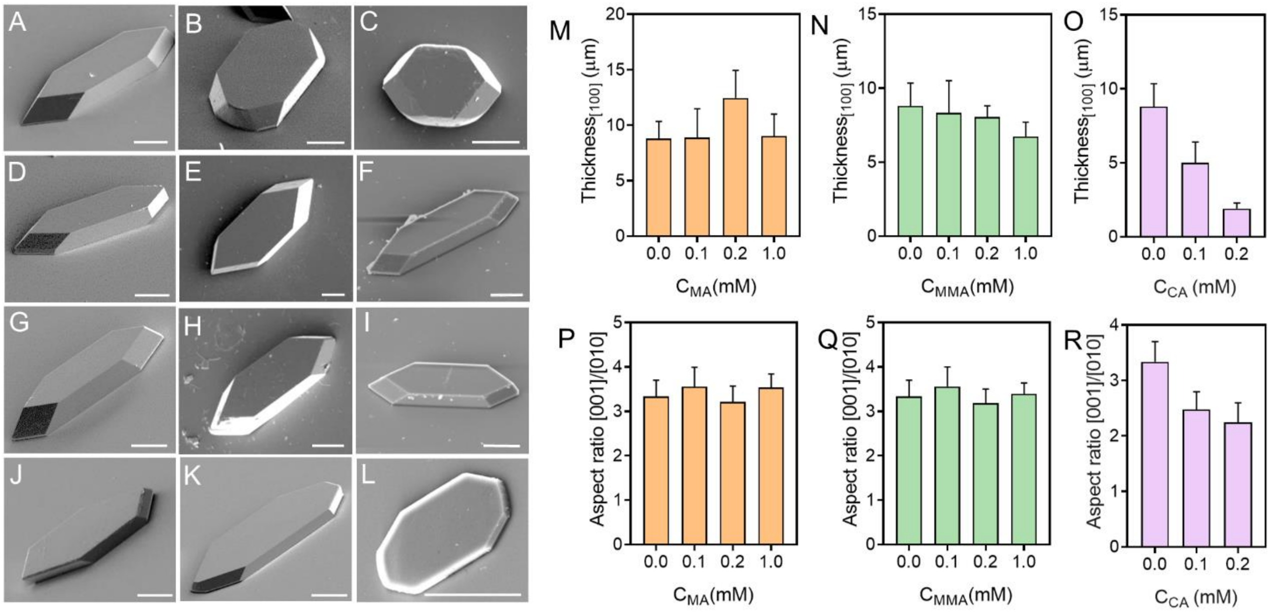

Figure S2. A-L) Scanning electron micrographs of COM crystals (A) in the absence of inhibitor and in the presence of (B) 0.1 mM TA, (C) 0.2 mM TA, (D) 0.1 mM MA, (E) 0.2 mM MA, (F) 1.0 mM MA, (G) 0.1 mM MMA, (H) 0.2 mM MMA, (I) 1.0 mM MMA, (J) 0.1 mM CA, (K) 0.2 Mm CA, and (L) 1.0 mM CA. All scale bars equal 20 μm. M-O) Comparison of COM [100] average thickness with inhibitor concentration, $C_{\text{inhibitor}}$. The average values of thickness were obtained from measurements of approximate 20 crystals from three separate batches. P-R) Changes in COM [001]/[010] aspect ratio with inhibitor concentration, $C_{\text{inhibitor}}$. A minimum of 150 crystals from three separate batches were measured to obtain an average [001]/[100] aspect ratio. Error bars equal one standard deviation in panels M-R.

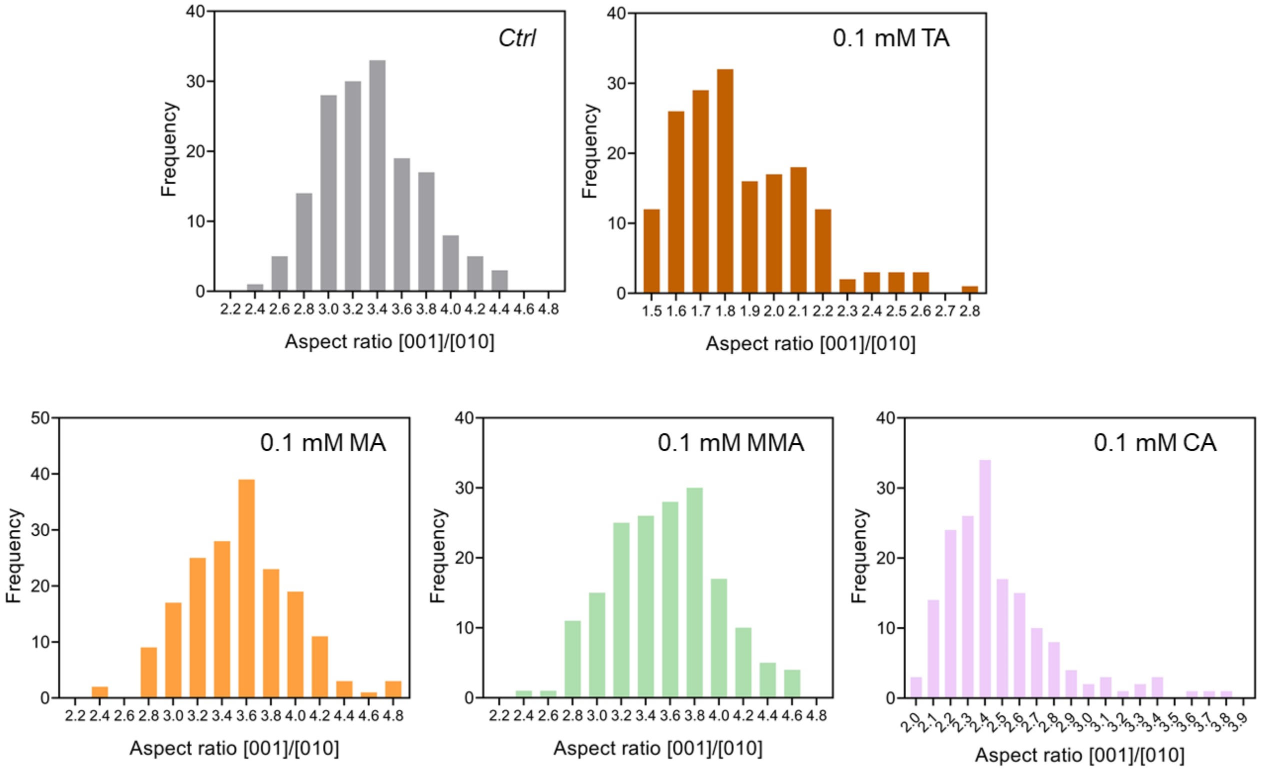

Figure S3. Quantitative analysis of the distribution of [001]/[010] aspect ratios of COM crystals collected from three separate batches. The aspect ratio is defined according to the relative dimensions of the crystal along the [001] and [010] directions. TA and CA presence resulted in a significantly increased frequency of crystals with low aspect ratio, indicating their influence on crystal morphology.

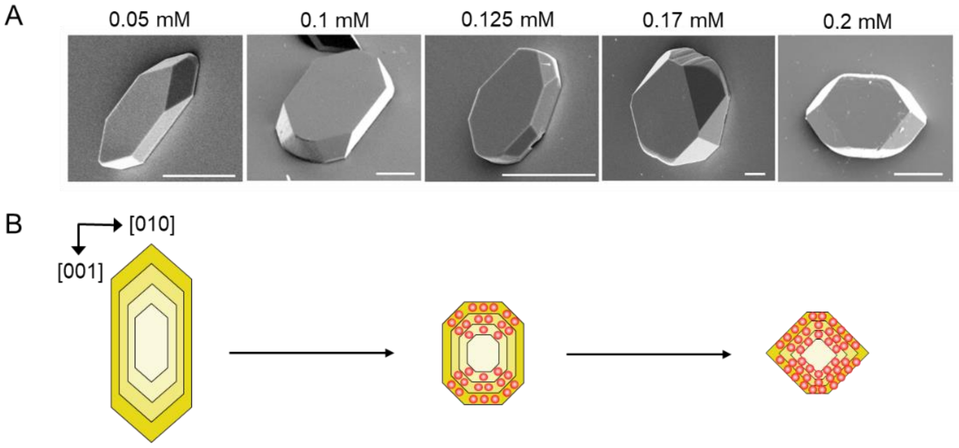

Figure S4. Impact of TA on COM crystal morphology. A) Scanning electron micrographs of COM crystals in the presence of 0.05 mM, 0.1 mM, 0.125 mM, 0.17 mM and 0.2 mM TA. All scale bars equal 20 μm. The COM crystal morphology, especially along the [001] direction, exhibited a notable change as the apical tips gradually became blunt with increasing TA concentration. B) Illustrations of the COM crystal habit and its modification by TA-induced growth inhibition of apical faces.

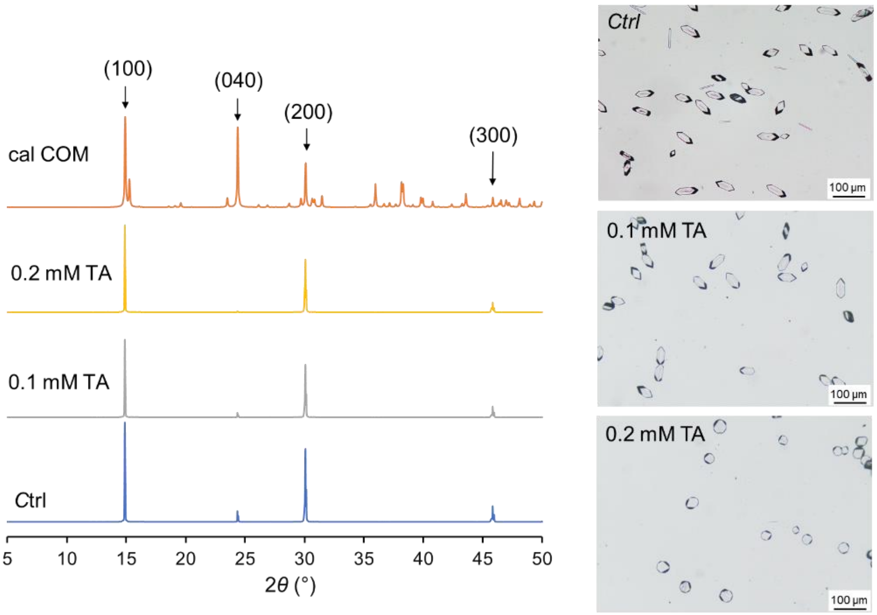

Figure S5. X-ray diffraction (XRD) spectra of COM crystals grown on the glass slide obtained in the bulk crystallization assays. From top to bottom, calculated XRD spectra based on the COM single crystal structure, experimental XRD spectra in the presence of 0.2 mM and 0.1 mM TA, as well as in the absence of inhibitor. In the XRD patterns, only peaks corresponding to {100} and {010} surfaces are observed, with {100} surfaces being predominant, revealing the basal surface as the (100) face, both in the absence and presence of tartronic acid. Notably, there is a significant decrease in the intensity of the (040) peak (representing {010} surface) with increasing TA concentration, consistent with the observed disappearance of the (010) surface in the presence of 0.2 mM TA.

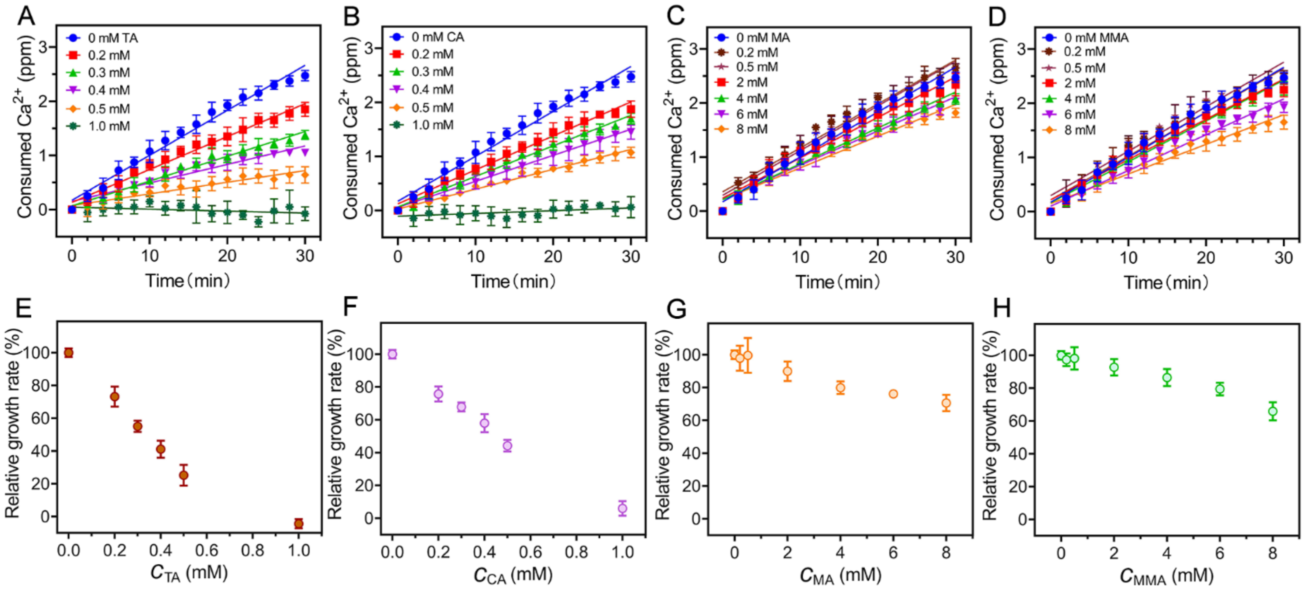

Figure S6. A-D) *In situ* ISE measurements of COM crystallization in the presence of (A) TA and (B) CA at concentration of 0-1.0 mM, and (C) MA and (D) MMA at concentrations of 0-8 mM. The *y-axis* represents the consumed quantity of free calcium ions in the solution during crystal growth. The slope

of each linear curve could be regarded as the rate of crystal growth. E-H) Relative growth rate of COM crystallization as a function of $C_{\text{inhibitor}}$. The relative growth rate of COM crystallization is obtained by comparing the growth rates in the presence of inhibitors to that in the absence of inhibitors. A minimum of four measurements were conducted for each data point. Error bars equal span two standard deviations. The unit of ppm is parts per million.

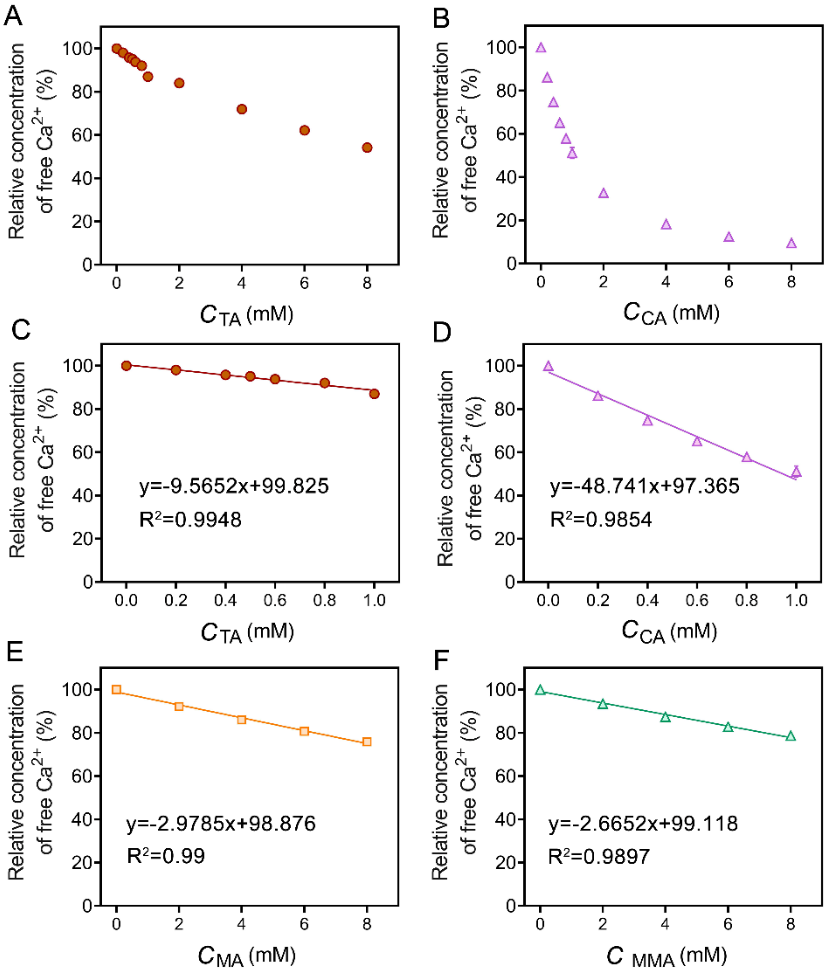

Figure S7. The relative concentration of free Ca^{2+} as a function of inhibitor concentration in the solution only containing $CaCl_2$ and inhibitors (the total concentration of Ca^{2+} is 0.5 mM). The significant reduction of free Ca^{2+} with increasing concentration of (B, D) CA indicates that CA exhibits a strong ability to complex free Ca^{2+} ions. Similar measurements for (A, C) TA, (E) MA, and (F) MMA reveal comparable and relatively weak complexing abilities with Ca^{2+}. Each data point is based on a minimum of three measurements. Linear regression analyses were performed to establish the relationship between inhibitor concentrations and the relative concentration of free Ca^{2+}. These analyses spanned concentration ranges from 0 to 1.0 mM for (C) TA and (D) CA, and from 0 to 8 mM for (E) MA and (F) MMA.

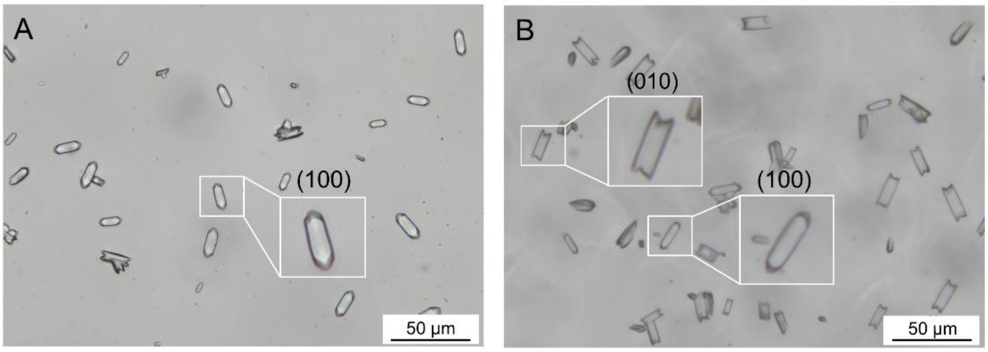

Figure S8. Representative optical micrographs of COM seeds growing in a microfluidic channel prepared (A) without (Method 1) and (B) with $NaHCO_3$ (Method 2). Most COM crystals prepared without $NaHCO_3$ have their (100) surface oriented upward. Crystals with (100) and (010) faces oriented upward were obtained in the presence of 4.04 mM $NaHCO_3$.

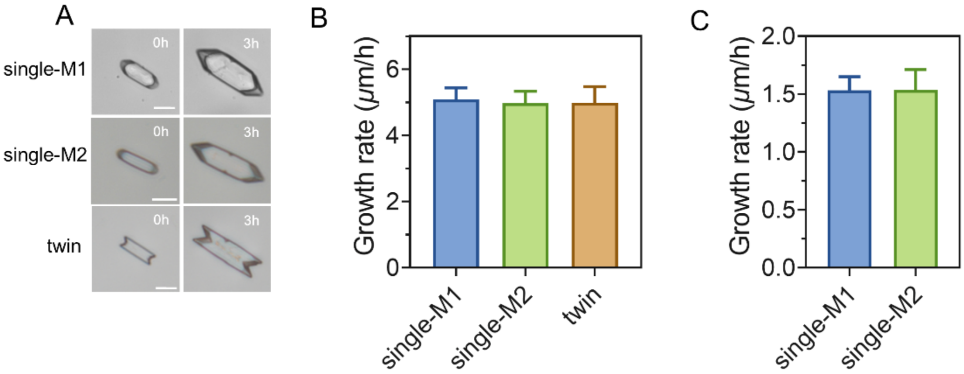

Figure S9. Growth rates of (A) COM crystals prepared using different solutions along the (B) [001] and (C) [010] directions in a microchannel under constant flow (0.5 ml h^{-1}) and supersaturation ratio S = 3.6. The labels “single-M1” and “single-M2” represent single crystals prepared by Methods 1 and 2, respectively. The label “twin” represents twin crystals prepared by Method 2. Notably, the growth rates along specific directions of COM crystals remain almost constant irrespective of the preparation method and the nature of the crystal (single or twin). A minimum of 50 crystals from at least three individual trials were measured to obtain the average growth rate in the [001] and [010] directions. Error bars equal one standard deviation in panels B and C.

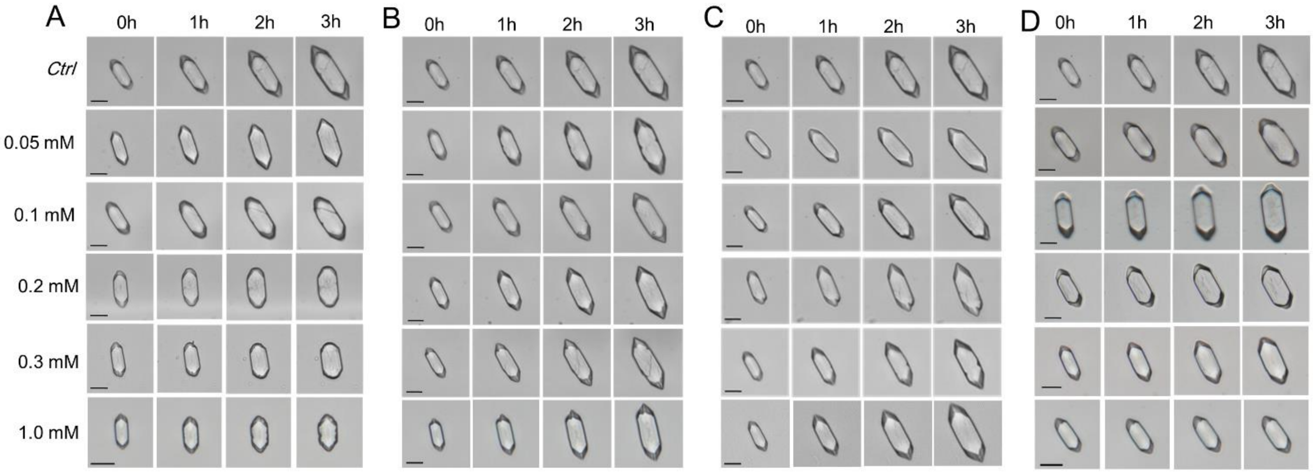

Figure S10. Time-elapsed optical micrographs demonstrating the effects of (A) TA, (B) MA, (C) MMA, and (D) CA on COM growth. Macroscopic growth rates of COM crystals were imaged in [001] and [010] directions. All scale bars are equal 10 μm. The flow rate is 0.5 ml h^{-1}. The introduction of TA significantly blunted COM crystals apical tips.

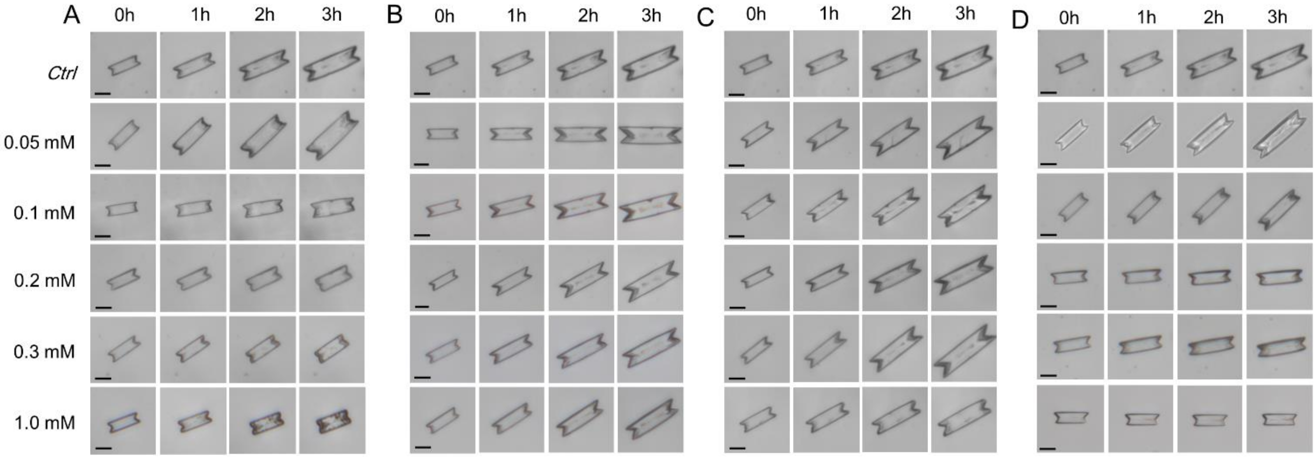

Figure S11. Time-elapsed optical micrographs demonstrating the effects of (A) TA, (B) MA, (C) MMA and (D) CA on COM growth. Macroscopic growth rates of COM crystals were imaged in [001] and [100] directions. All scale bars equal 10 μm. The flow rate is 0.5 ml h^{-1}.

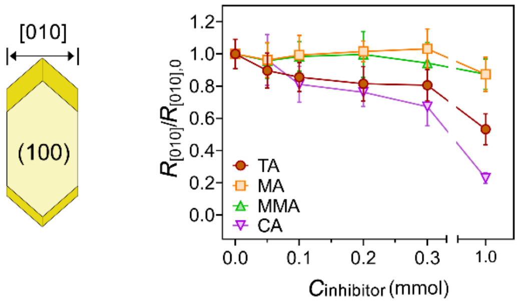

Figure S12. Relative growth rate in the [010] direction of COM crystals (width) as a function of inhibitor concentration. Symbols are the averages of at least three measurements (around 15 crystals for each measurement), and error bars span two standard deviations.

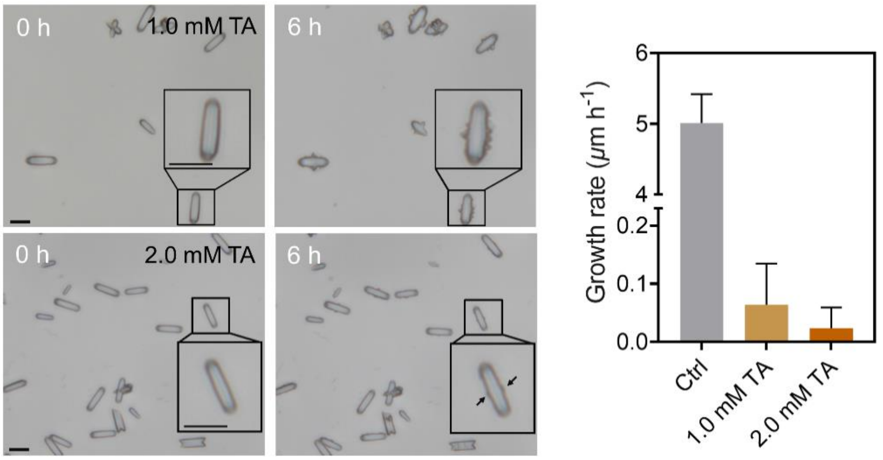

Figure S13. COM crystal growth in the presence of 1.0 mM and 2.0 mM TA under flow conditions exhibit protrusions on the (010) surfaces. The growth rate of protrusions decreased with increasing TA concentration. All scale bars equal 10 μm.

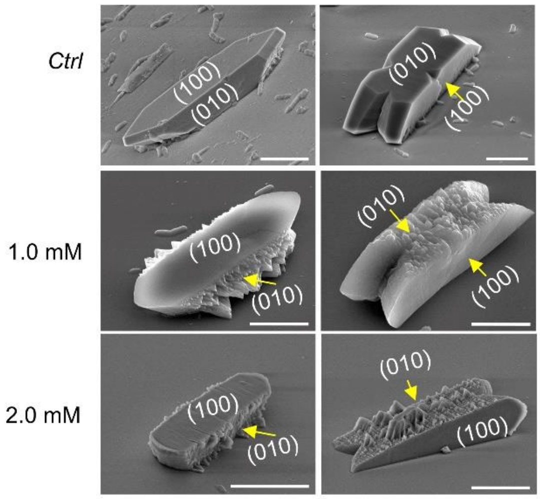

Figure S14. SEM images of COM crystals after growing under a flow condition for 6 hours in the absence (*Ctrl*) and presence of 1.0 mM and 2.0 mM TA. Sword-like protrusions were observed on COM

(010) surfaces (indicated by yellow arrows) when crystals were grown in the presence of high concentrations of TA. All scale bars equal 5 μm.

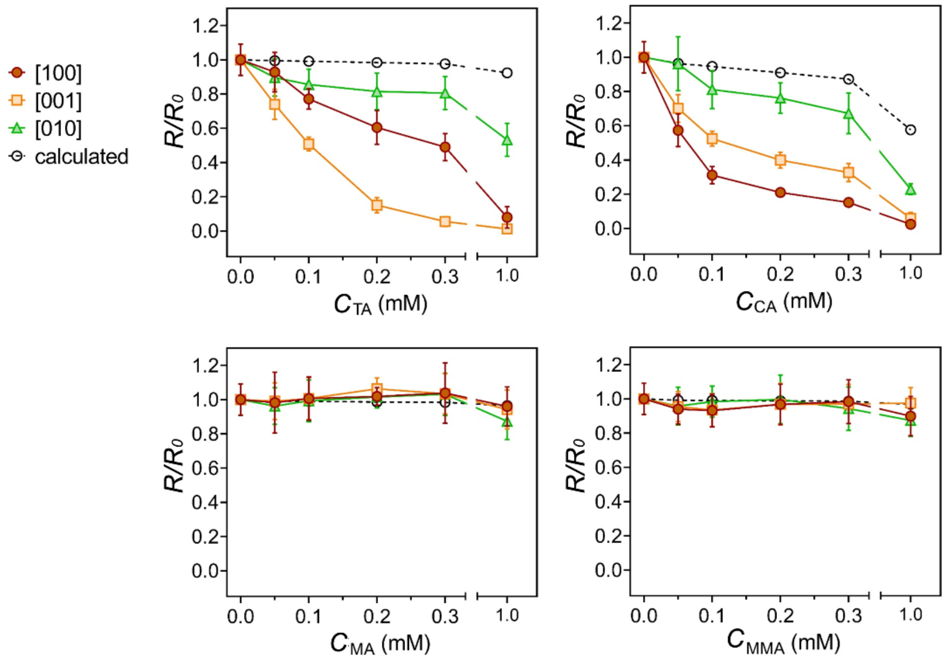

Figure S15. Relative growth rate R/R_0 in the three principal directions of COM crystals as a function of inhibitor concentration. The black circles represent the predicted relative growth rates (R/R_0) on the basis of decreased free Ca^{2+} ion concentration (i.e., supersaturation) resulting from complexing by inhibitors. This estimation presumes the kinetic coefficient of crystal growth is independent of inhibitors and the predicted relative step velocity v/v_0 was obtained using equation S4. The supersaturation changes in the present of inhibitors are determined by the linear fitting equation in Figure S7.

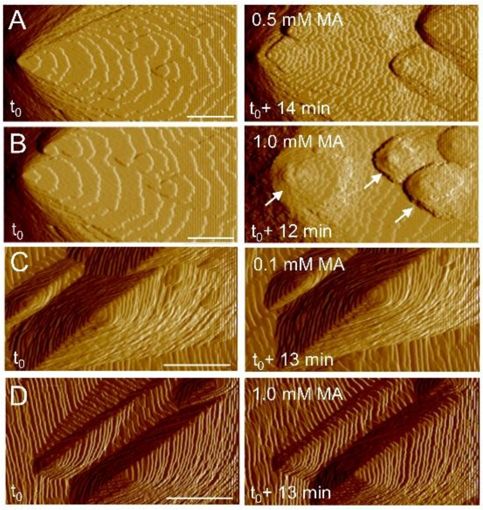

Figure S16. The impact of malonic acid (MA) on COM surface growth. AFM deflection mode images of COM (A, B) (100) and (C, D) (010) surfaces during *situ* measurements in a supersaturated solution (S = 4.1) at (C) 0.1 mM MA, (A) 0.5 mM MA, and (B, D) 1.0 mM MA. The left images were captured

at initial scanning times, and those on the right were taken after ca. 13 minutes of continuous imaging. With increasing MA concentration, the [001] step became rough and the interstep distances decreased. Arrows in panel (B) highlight rounded hillocks on the COM (100) surface in the presence of 1.0 mM MA. In contrast, MA had little effect on the (021) and ($12\bar{1}$) steps. All scale bars equal 1.0 μm.

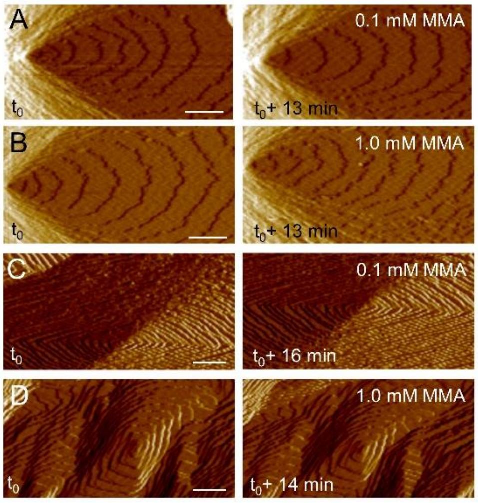

Figure S17. Impact of methylmalonic acid (MMA) on COM surface growth. AFM deflection mode images of COM (A, B) (100) and (C, D) (010) surfaces during *in situ* measurements in supersaturated solution (S = 4.1) at (A, C) 0.1 mM MMA and (B, D) 1.0 mM MMA. The left images were captured at initial scanning times, and those on the right were taken after ca. 13 minutes of continuous imaging. MMA exhibited little effect on the growth of both (100) and (010) surfaces. All scale bars equal 0.5 μm.

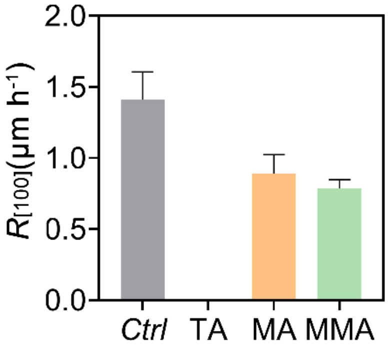

Figure S18. The growth rate $R_{[100]}$ of COM crystals in the microfluidic channels along the [100] direction in the absence (*Ctrl*) and presence of 2.0 mM TA, MA, and MMA. The experimental method employed is consistent with that described in Section 2.3 of the manuscript. Notably, 2.0 mM TA completely suppressed the growth, while the effects of MA and MMA were relatively weak. Error bars represent one standard deviation.

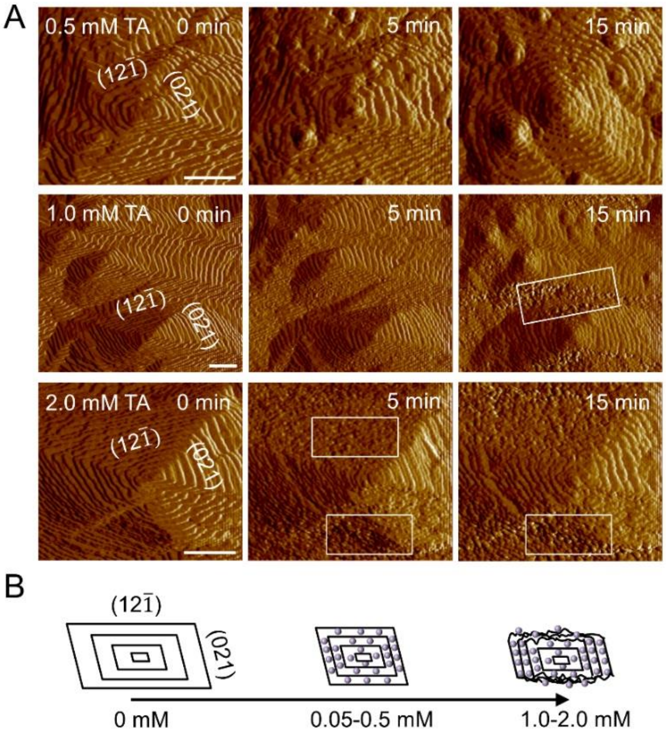

Figure S19. Impact of TA on COM (010) surface growth. A) Time-elapsed *in situ* AFM deflection mode images of COM (010) surfaces in the presence of 0.5 mM, 1.0 mM, and 2.0 mM TA (from top to bottom). At low concentrations of TA (≤0.5 mM), the initially rectangular growth hillocks are transformed into a square shape due to a preferred inhibition of (021) step advancement. Introducing 1.0 mM or higher concentration of TA, the step edges became serrated within 15 minutes. Notably, (12$\bar{1}$) steps located away from the spiral centers became significantly coarsened and indistinguishable (in white square). All scale bars equal 0.5 μm. B) Idealized scheme of TA interacting with growth hillocks on the COM (010) surface.

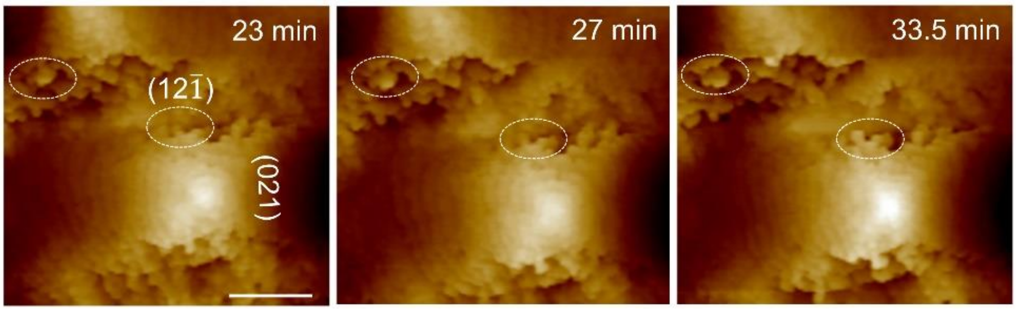

Figure S20. Time-elapsed *in situ* AFM height images of a COM (010) surface in the presence of 1.0 mM TA. The (12$\bar{1}$) steps away from the spiral center became significantly roughened with small protrusions, and the advancement of such protrusions was almost limited to finite regions (in dashed white circle). The scale bar equals 0.5 μm.

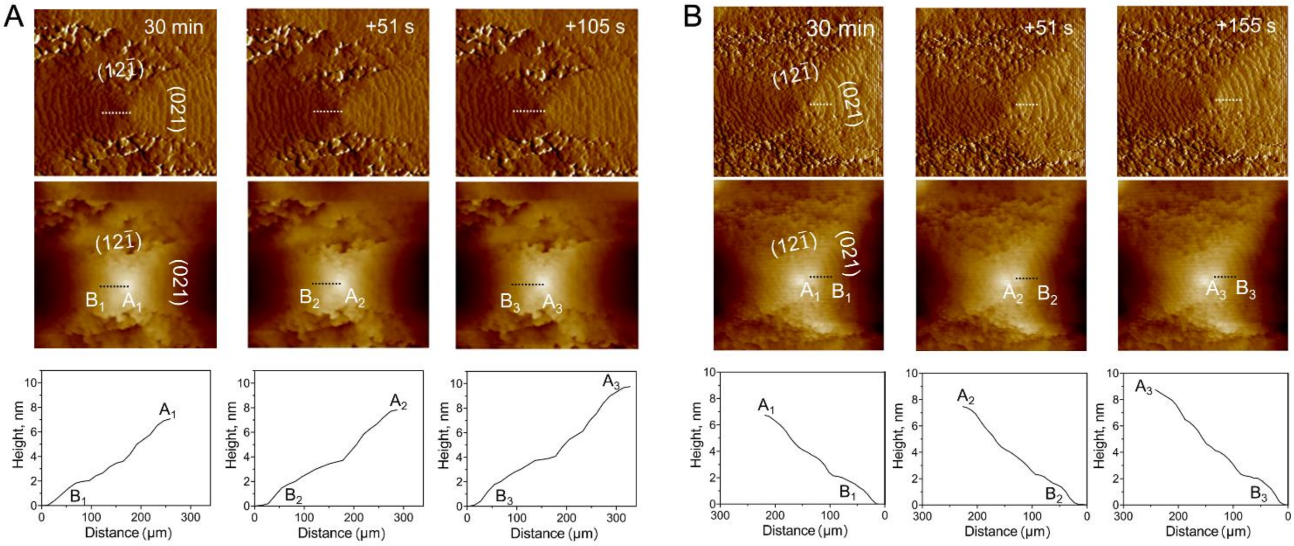

Figure S21. Time-elapsed *in situ* AFM deflection mode images of COM (010) surface step growth in the presence of (A) 1.0 mM and (B) 2.0 mM TA after 30 minutes. The (12$\bar{1}$) steps were significantly disrupted in the presence of a high concentration of TA, but the spiral growth of hillocks persisted, resulting in vertical increments in (010) surfaces. In the presence of 1.0 mM TA, the height from site B to site A increased from 7.0 to 9.7 nm within 105 seconds. As the TA concentration increased to 2 mM, the vertical increments in (010) surfaces reduced, with the height from site B to site A increasing from 6.7 to 8.7 nm within 155 seconds. All scale bars equal 0.5 μm.

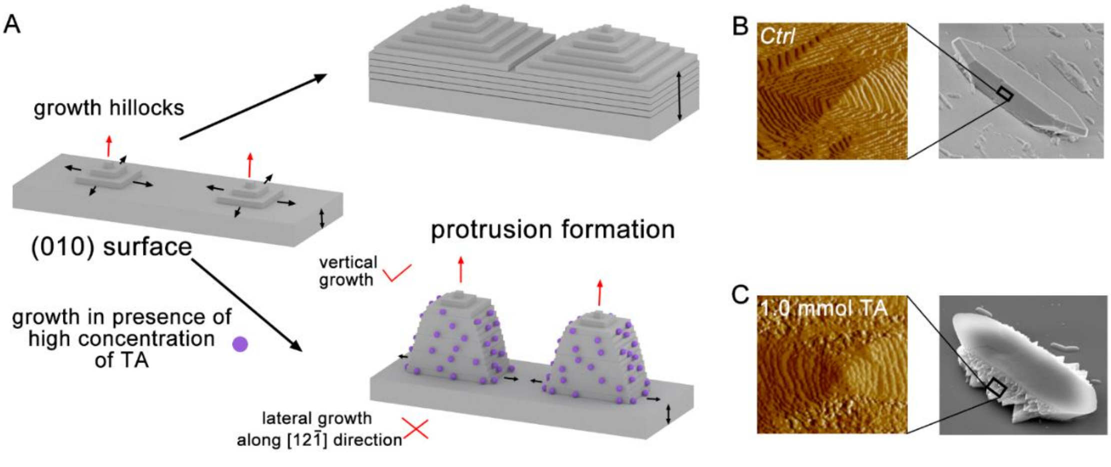

Figure S22. A) Idealized illustration of protrusion formation on COM (010) surfaces in the presence of TA. The crystal growth along the [010] direction results from the lateral and vertical growth of hillocks on the (010) plane. B, C) The AFM results reveal that in the presence of high concentration of TA, the (12$\bar{1}$) steps located away from the hillock centers became significantly coarsened compared to those in the absence of TA, and the advancement of these steps almost ceased while new steps were continuously generated from the dislocation source. The vertical increments due to spiral growth, coupled with the inhibition of lateral hillock advancement due to the destruction of (12$\bar{1}$) steps by the inhibitor, resulted

in the formation of sword-like protrusions on (010) surfaces at high TA concentration. These protrusions are observable in the macroscopic crystal morphology under conditions of elevated TA concentration, as shown in the SEM image in panel C.

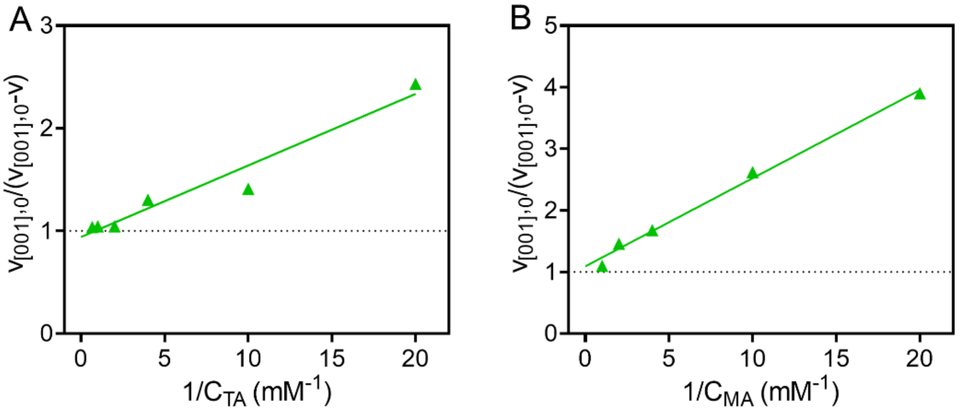

Figure S23. The correlation between the step velocity of the [001] step and the inhibitor concentration for measurements in the presence of (A) TA and (B) MA. Data are presented in linearized coordinates $v_0(v_0\text{-}v)^{-1}$ and the $C_{\text{inhibitor}}^{-1}$. The solid straight line represents the best linear regression fit to the data. The approximately linear correlation between $v_0(v_0\text{-}v)^{-1}$ and the $C_{\text{inhibitor}}^{-1}$ is a characteristic of kink blocking.[7]

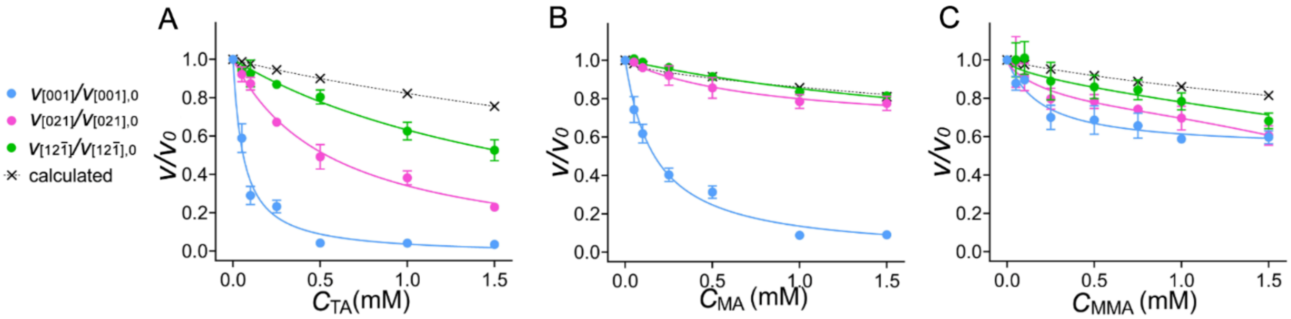

Figure S24. Step velocity v in the presence of inhibitor scaled by the control in the absence of inhibitor, v_0, as a function of (A) TA, (B) MA, and (C) MMA concentration. Green, blue, and magenta circles represent step advancement in the $[12\bar{1}]$, [021], and [001] direction, respectively. Symbols are the average of at least five measurements, and error bars span two standard deviations. The black crosses represent the predicted relative step velocity (v/v_0) using equation S4 on the basis of the decrease in free Ca^{2+} resulting from complexing by inhibitors. This estimation presumes the kinetic coefficient of crystal growth is independent of inhibitors and the supersaturation changes in the present of inhibitors are determined by the linear fitting in Figure S7.

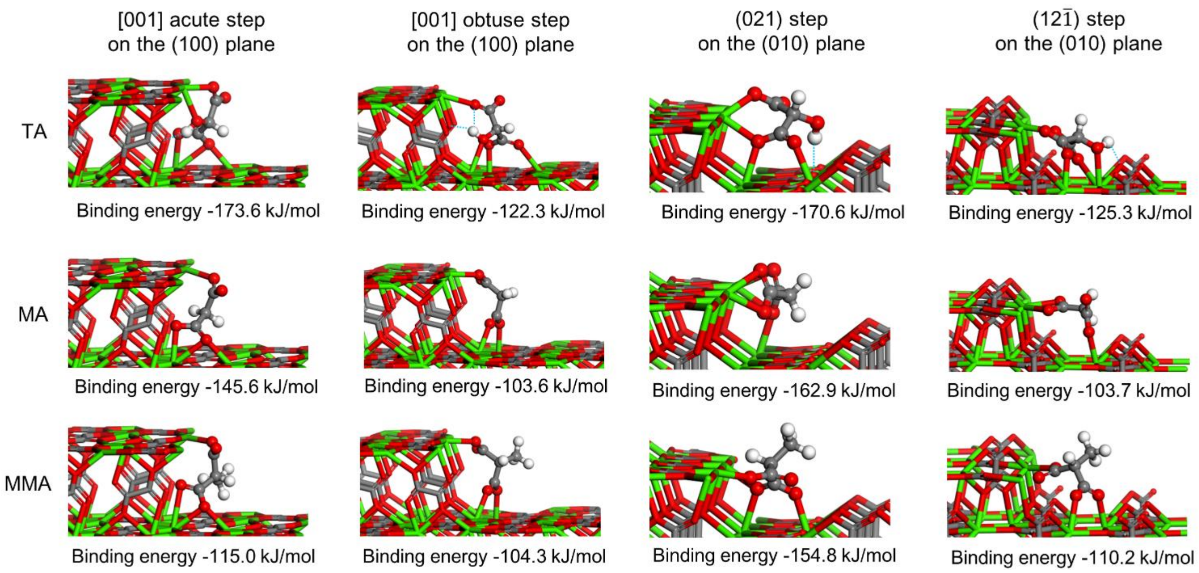

Figure S25. DFT calculation of modifiers binding to COM [001] acute and obtuse steps on the (100) surface, and (021) and (12$\bar{1}$) steps on the (010) surfaces. The structural conformation of fully deprotonated TA (top), MA (middle), and MMA (bottom) molecules binding to COM [001], (021) and (12$\bar{1}$) steps were optimized. In these calculations, the surfaces are kept frozen (unrelaxed). Atoms are colored to represent hydrogen (white), carbon (grey), oxygen (red), and calcium (green).

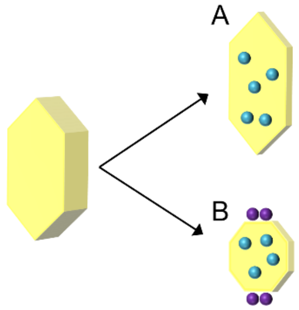

Figure S26. Illustration of the potential synergy between endogenous inhibitors and TA. A) Endogenous inhibitors (colored blue) tend to preferentially bind to the COM (100) surface.[8] B) In the presence of both endogenous inhibitors and TA (colored purple), the latter inhibits the growth of apical faces, and endogenous modifiers inhibit the basal (100) surface. This cooperative action may exert a synergetic influence on the inhibition of bulk crystallization.

Supplementary Tables

Table S1. List of primer sequences used for the real-time qPCR analysis

Name	(5'-3')	Reverse primer (5'-3')
KIM-1	Forward	ACAGTGGTCTGTATTGTTGTCGAGTG

	Reverse	TGTCACAGTGCCATTCCAGTCTG
OPN	Forward	GACCATGAGATTGGCAGTGAT
	Reverse	CTGTAGGGACGATTGGAGTGA
NGAL	Forward	CAGAAGGCAGCTTTACGATGT
	Reverse	AACTGGTTGTAGTCCGTGGTG
MCP-1	Forward	TCAAGAGAGAGGTCTGTGCTG
	Reverse	GGTGGTTGTGGAAAAGGTAGT
GAPDH	Forward	GAGAGTGTTTCCTCGTCCCGTAG
	Reverse	GCCTCACCCCATTTGATGTTAGT

Supplementary Movies

Movie S1. Time-elapsed sequence of AFM deflection mode images depicting the growth of hillocks on a COM (100) surface in supersaturated CaOx solution (S = 4.1). Continuous imaging is initially performed in the absence of TA (time t = 0 to 1.8 minutes), followed by the addition of the same growth solution containing C_{TA} = 0.1 mM. Under the influence of TA, the [001] step roughened, the inter-step distance decreased, and the hillocks became more rounded. The total imaging time for the *in situ* AFM video is 13.5 minutes.

Movie S2. Time-elapsed sequence of AFM deflection mode images depicting the growth of hillocks on a COM (100) surface in supersaturated CaOx solution (S = 4.1). Continuous imaging is initially performed in the absence of MA (time t = 0 to 2.8 minutes), followed by the addition of the same growth solution containing C_{MA} = 0.1 mM. In the presence of MA, the [001] step became serrated with decreasing inter-step distance. The total imaging time for the *in situ* AFM video is 14.6 minutes.

Movie S3. Time-elapsed sequence of AFM deflection mode images depicting the growth of hillocks on a COM (010) surface in supersaturated CaOx solution (S = 4.1). Continuous imaging is initially performed in the absence of TA (time t = 0 to 4 minutes), followed by the addition of the same growth solution containing C_{TA} = 1.0 mM. When 1.0 mM TA was introduced, the step edges became serrated within 10 minutes. Notably, the $(12\bar{1})$ steps farther away from the hillock centers became significantly coarsened with small protrusions, and the advancement of steps almost ceased. The total imaging time for the *in situ* AFM video is 13.5 minutes.

Movie S4. Time-elapsed sequence of AFM deflection mode images depicting the growth of hillocks on a COM (010) surface in supersaturated CaOx solution (S = 4.1). Continuous imaging is performed after

the introduction of 1.0 mM TA for approximately 30 minutes. The $(12\bar{1})$ steps farther away from the hillock centers became significantly coarsened and the advancement of steps almost ceased; however, new steps are constantly produced from the hillock centers via spiral growth.

Supplementary References

[1] a) L. Wang, S. R. Qiu, W. Zachowicz, X. Guan, J. J. D. Yoreo, G. H. Nancollas, J. R. Hoyer, *Langmuir* **2006**, *22*, 7279; b) J. Streit, L.-C. Tran-Ho, E. Königsberger, *Monatsh. Chem.* **1998**, *129*, 1225.

[2] P. G. Vekilov, *Cryst. Growth Des.* **2007**, *7*, 2796.

[3] a) A. B. Nair, S. Jacob, *JBCP* **2016**, *7*, 27; b) A. Nair, M. A. Morsy, S. Jacob, *Drug Dev. Res.* **2018**, *79*, 373; c) FDA, Guidance for Industry Estimating the Maximum Safe Starting Dose in Initial Clinical Trials for Therapeutics in Adult Healthy Volunteers **2005**.

[4] a) R. Phillips, V. S. Hanchanale, A. Myatt, B. Somani, G. Nabi, C. S. Biyani, *Cochrane Database Syst. Rev.* **2015**, *2015*, CD010057; b) T. Soyygür, A. Akbay, S. Küpeli, *J. Endourol.* **2002**, *16*, 149; c) M. R. Robinson, V. A. Leitao, G. E. Haleblian, C. D. Scales, Jr., A. Chandrashekar, S. A. Pierre, G. M. Preminger, *J. Urol.* **2009**, *181*, 1145; d) D. S. Goldfarb, F. Modersitzki, J. R. Asplin, L. Nazzal, *Urolithiasis* **2023**, *51*, 96; e) S. Doizi, J. R. Poindexter, M. S. Pearle, F. Blanco, O. W. Moe, K. Sakhaee, N. M. Maalouf, *J. Urol.* **2018**, *200*, 1278; f) P. Barcelo, O. Wuhl, E. Servitge, A. Rousaud, C. Y. Pak, *J. Urol.* **1993**, *150*, 1761.

[5] B. Lojanapiwat, M. Tanthanuch, C. Pripathanont, S. Ratchanon, S. Srinualnad, T. Taweemonkongsap, S. Kanyok, S. Lammongkolkul, *Int. Braz. J. Urol.* **2011**, *37*, 611.

[6] E. Cicerello, F. Merlo, G. Gambaro, L. Maccatrozzo, A. Fandella, B. Baggio, G. Anselmo, *J. Urol.* **1994**, *151*, 5.

[7] a) J. Chung, M. G. Taylor, I. Granja, J. R. Asplin, G. Mpourmpakis, J. D. Rimer, *Cryst. Growth Des.* **2018**, *18*, 5617; b) W. Ma, J. F. Lutsko, J. D. Rimer, P. G. Vekilov, *Nature* **2020**, *577*, 497.

[8] a) S. R. Qiu, A. Wierzbicki, C. A. Orme, A. M. Cody, J. R. Hoyer, G. H. Nancollas, S. Zepeda, J. J. D. Yoreo, *Proc. Natl. Acad. Sci. U.S.A.* **2004**, *101*, 1811; b) S. Farmanesh, S. Ramamoorthy, J. Chung, J. R. Asplin, P. Karande, J. D. Rimer, *J. Am. Chem. Soc.* **2014**, *136*, 367; c) J. Chung, I. Granja, M. G. Taylor, G. Mpourmpakis, J. R. Asplin, J. D. Rimer, *Nature* **2016**, *536*, 446.
